# Human selenocysteine synthase, SEPSECS, has evolved to optimize binding of a tRNA-based substrate

**DOI:** 10.1093/nar/gkae875

**Published:** 2024-10-10

**Authors:** Anupama K Puppala, Dylan Sosa, Jennifer Castillo Suchkou, Rachel L French, Malgorzata Dobosz-Bartoszek, Kaitlyn A Kiernan, Miljan Simonović

**Affiliations:** Department of Biochemistry and Molecular Genetics, University of Illinois at Chicago, Chicago, IL 60607, USA; Department of Ecology & Evolution, University of Chicago, Chicago, IL 60637, USA; Department of Biochemistry and Molecular Genetics, University of Illinois at Chicago, Chicago, IL 60607, USA; Department of Biochemistry and Molecular Genetics, University of Illinois at Chicago, Chicago, IL 60607, USA; Department of Biochemistry and Molecular Genetics, University of Illinois at Chicago, Chicago, IL 60607, USA; Department of Biochemistry and Molecular Genetics, University of Illinois at Chicago, Chicago, IL 60607, USA; Department of Biochemistry and Molecular Genetics, University of Illinois at Chicago, Chicago, IL 60607, USA

## Abstract

The evolution of the genetic code to incorporate selenocysteine (Sec) enabled the development of a selenoproteome in all domains of life. *O*-phosphoseryl-tRNA^Sec^ selenium transferase (SepSecS) catalyzes the terminal reaction of Sec synthesis on tRNA^Sec^ in archaea and eukaryotes. Despite harboring four equivalent active sites, human SEPSECS binds no more than two tRNA^Sec^ molecules. Though, the basis for this asymmetry remains poorly understood. In humans, an acidic, C-terminal, α-helical extension precludes additional tRNA-binding events in two of the enzyme monomers, stabilizing the SEPSECS•tRNA^Sec^ complex. However, the existence of a helix exclusively in vertebrates raised questions about the evolution of the tRNA-binding mechanism in SEPSECS and the origin of its C-terminal extension. Herein, using a comparative structural and phylogenetic analysis, we show that the tRNA-binding motifs in SEPSECS are poorly conserved across species. Consequently, in contrast to mammalian SEPSECS, the archaeal ortholog cannot bind unacylated tRNA^Sec^ and requires an aminoacyl group. Moreover, the C-terminal α-helix 16 is a mammalian innovation, and its absence causes aggregation of the SEPSECS•tRNA^Sec^ complex at low tRNA concentrations. Altogether, we propose SEPSECS evolved a tRNA^Sec^ binding mechanism as a crucial functional and structural feature, allowing for additional levels of regulation of Sec and selenoprotein synthesis.

## Graphical Abstract

**Graphical Abstract:**
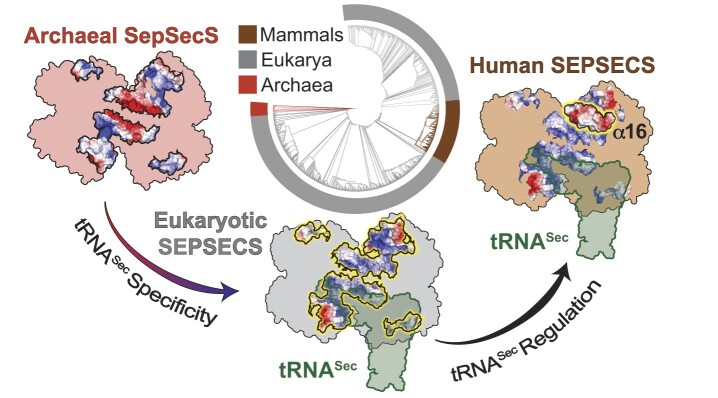

## Introduction

Selenium is an indispensable antioxidant micronutrient for organisms in all domains of life (1,2). The primary biological metabolite of selenium is selenocysteine (Sec), the 21st proteinogenic amino acid. Though the typical selenoproteome is of limited size, its members are critical for the health, development, and survival of the organism. In humans, two dozen selenoproteins help maintain cellular redox potential (3–5), mitigate oxidative damage (6–8) and regulate thyroid hormone homeostasis and metabolic rate (9,10). Selenoenzymes such as thioredoxin reductase, glutathione peroxidase, and methionine sulfoxide reductase replenish important antioxidants and safeguard against oxidative damage (6–8). The replacement of Sec with chemically similar Cys or Ser often diminishes the catalytic prowess of such selenoenzymes (11–13). Consequently, mutations in genes encoding selenoproteins or proteins facilitating selenoprotein synthesis generally cause multi-organ and/or systemic disorders (14).

However, neither the biosynthetic pathway for free Sec nor a putative Sec-tRNA synthetase ever evolved (15–17). Rather, accurate translation of selenoprotein genes relies on a set of specific anabolic enzymes and tRNA^Sec^ along with a nuanced mechanism of co-translational Sec insertion, dependent on recoding of an in-frame UGA codon (18). tRNA^Sec^ possesses a series of structural elements distinct from other canonical elongator tRNAs, which help ensure high specificity and fidelity of the Sec biosynthetic cycle (19). Generally, an elongator tRNA adopts a canonical 7/5 configuration between the acceptor and TΨC arms. By contrast, bacterial tRNA^Sec^ (btRNA^Sec^) adopts an 8/5 configuration (20), while the archaeal and eukaryotic orthologs fold into a 9/4 arrangement (21). Each of these tRNA^Sec^ variants also features elongated D- and variable arms that serve as additional identity elements (22,23). Nonetheless, in all organisms Sec synthesis initiates upon seryl-tRNA synthetase coupling Ser to the non-cognate tRNA^Sec^ (24). Following serylation, selenocysteine synthase (SelA) directly converts Ser-to-Sec in bacteria (25,26). However, archaea and eukaryotes employ *O*-phosphoseryl-tRNA^Sec^ kinase (PSTK) to first phosphorylate the hydroxyl group on Ser (27,28). Finally, in the terminal synthetic reaction, *O*-phosphoseryl-tRNA^Sec^ selenium transferase (SepSecS) substitutes the phosphoryl group for the selenol group in a reaction that requires mono-selenophosphate and a pyridoxal phosphate (PLP) co-factor (29,30).

Although SelA and SepSecS belong to the family of Fold Type I PLP-dependent enzymes, they act on different tRNA-based substrates, have distinct catalytic mechanisms, and occupy separate phylogenetic branches (31). In this work, we use SepSecS to refer to the enzyme generally and for archaeal species, whereas for eukaryotic species of the enzyme we use SEPSECS, following the International Protein Nomenclature Guidelines. A pentamer of SelA dimers form a ring structure that binds 10 btRNA^Sec^ molecules from the D-arm side, yielding a stoichiometric complex (26). Conversely, archaeal and eukaryotic holo SepSecS exists as a dimer of dimers with four equivalent monomers (32). Binding of tRNA^Sec^ to human SEPSECS generates an asymmetric complex, comprised of catalytic and non-catalytic SEPSECS protomers and with only one or two of the four sites bound (33). To accommodate binding, helices α1 and α9 of one protomer engage with the 13 bp acceptor-TΨC helix and variable arm of tRNA^Sec^, respectively (34). Simultaneously, α14 and α15 of the other protomer bind to the tip of the acceptor arm in a cross-dimer fashion and help position the G73 discriminator base and the aminoacylated CCA-end toward the active site crevice (29,34,35). While the acceptor and TΨC arms constitute the predominant identity elements mediating tRNA^Sec^ recognition, the variable arm is not a major identity element but rather helps discriminate against incorrect tRNA binding (34). Notably, both tRNA^Sec^ molecules always bind to the same SEPSECS dimer/protomer and other arrangements have not been observed (29,33,34). Recently, we have shown that the newly discovered C-terminal α16, encompassing residues Glu477-Leu493, may regulate the observed structural asymmetry of the SEPSECS•tRNA^Sec^ binary complex by defining the catalytic and non-catalytic protomers (34). Our results revealed that tRNA^Sec^ docking caused an otherwise disordered α16 helix to stabilize within the tRNA-binding pockets of the neighboring monomers (34). Consequently, α16 interacts with α14 and α15 of the same monomer, thus blocking the active sites and additional tRNAs from binding to the now defined non-catalytic dimer.

However, the C-terminal region of eukaryotic SEPSECS is not well-characterized and its function has remained elusive. Our current understanding of the SEPSECS C-terminus largely stems from studies of individuals afflicted by chronic autoimmune hepatitis (AIH), a chronic inflammatory liver disease (36). These AIH patients produced autoantibodies that target residues 450–490 in SEPSECS and precipitated a SEPSECS•tRNA^Sec^ complex from mammalian cell extracts (37–39). Thus, questions about the origin, conservation, and role of α16 in tRNA binding, catalysis, and its potential impact on Sec and selenoprotein synthesis remain unanswered. To that end, we used structural, biophysical, and phylogenetic methods to show that the appended C-terminus in human SEPSECS plays a pivotal role in defining and stabilizing the quaternary structure of the terminal Sec-synthetic complex. Upon tRNA binding, electrostatic interactions between acidic residues of α16 and basic residues of α14 and α15 anchor α16 into the tRNA-binding pocket of the non-catalytic protomer. In the absence of the C-terminal tail, tRNA binding induces aggregation of the binary complex in a dose-dependent manner with a greater magnitude of aggregation at lower tRNA concentrations. Whereas mammalian SEPSECS has evolved positively charged surfaces to stabilize the negatively charged RNA and engage tRNA nucleotides, the archaeal orthologs likely employ a mechanism of Sep-tRNA^Sec^ binding that primarily relies on the aminoacyl moiety and not on tRNA^Sec^ itself. Only some vertebrate species have evolved an acidic α-helix in the C-terminus of SEPSECS, which does not appear to be vertically inherited through the vertebrate lineages. Rather, the human α16 sequence appears to be unique to mammalian SEPSECS. Altogether, these findings suggest that human SEPSECS has evolved to recognize and stabilize the tRNA portion of the phosphoseryl-tRNA^Sec^ substrate.

## Materials and methods

### Cloning of SepSecS, PSTK, tRNA^Sec^

Human SEPSECS and *Methanococcus maripaludis* (MMP) SepSecS were each cloned into a pET15b vector, while human, MMP, and *Escherichia coli* tRNA^Sec^ were each cloned into a pUC19 vector, as previously described (29,40). To generate Δ470 SEPSECS, we performed PCR using the following mutagenic primers to mutate Asp470 to a stop codon in human WT SEPSECS on the pET15b vector (pET15b-hWTSSS) using the QuikChange II XL Site-Directed Mutagenesis Kit (Agilent).

Forward:

5′-GTAAGAAAAGAACGAAGTAAAGAGAGTTAGGACAATTATGACAAAACTGAAGATGTG-3′

Reverse:

5′-CACATCTTCAGTTTTGTCATAATTGTCCTAACTCTCTTTACTTCGTTCTTTTCTTAC-3′

Sanger sequencing confirmed the presence of the desired mutations.

For the SEPSECS *E. coli* complementation assay, a codon-optimized PSTK from *Methanocaldococcus jannaschii* (MjPSTK) was cloned into a pACYCDuet-1 vector as previously described (34). Subsequently, either the pET15b empty vector or a pET15b SEPSECS construct was transformed into the Δ*selA* JS2(DE3) strain to generate strains possessing dual pET and pACYC vectors.

### Expression and purification of SepSecS

For large-scale expression of SepSecS, the pET15b vector carrying either the human WT, MMP WT or Δ470 human SEPSECS was transformed into C41(DE3) cells and plated onto LB agar plates with ampicillin (100 μg/ml). A single colony was selected and grown to density overnight in LB. This starter culture was used to inoculate 4 l of LB media with ampicillin (100 μg/ml) at a ratio of 1:100. The 4 l culture grew at +37°C until the cells reached log phase, at an optical density of 0.6–0.8 at 600 nm (OD_600_). To induce protein expression, IPTG was added to a final concentration of 0.5 mM. The cultures then continued to grow at +15°C for 18–20 h. The cells were harvested by spinning the cultures in a centrifuge at 5000 rpm. The pelleted cells were flash-frozen using liquid nitrogen and stored at −80°C.

For human SEPSECS purification, the cells were lysed by sonication in lysis buffer (50 mM HEPES, pH 8.0, 300 mM NaCl, 10% (v/v) glycerol, 10 mM imidazole, 10 μM PLP and 3 mM βMe) supplemented with a protease inhibitor tablet (Roche), using 4 ml of lysis buffer per gram of cell pellet. To separate the soluble cell lysate from the cell debris, the sonicated suspension was centrifuged at 27 000 × *g* for 45 min. The soluble lysate was then loaded onto an FF Crude Ni^2+^-affinity column (Cytiva) equilibrated with lysis buffer. The column was rinsed with 200 ml of lysis buffer, followed by 200 ml of wash buffer (lysis buffer with 60 mM imidazole). Finally, affinity bound SEPSECS was eluted with elution buffer (lysis buffer with 350 mM imidazole). The SEPSECS eluate was loaded onto a S200 16/600 Superdex (Cytiva) size-exclusion chromatography (SEC) column that was pre-equilibrated with 20 mM HEPES, pH 8.0, 200 mM NaCl, 5% (v/v) glycerol, 10 μM PLP and 0.5 mM tris(2-carboxyethyl) phosphine (TCEP). For MMP SepSecS, purification was performed with a Tris-based buffer (20 mM Tris, pH 8.0) instead of HEPES. For all SepSecS species, the purified sample was concentrated using a 30 kDa MWCO centrifugal concentrator (Amicon), filtered through a 0.22 μm filter, flash-frozen in liquid nitrogen, and stored at −80°C. Concentration and purity of the purification process was assessed by SDS-PAGE.

### *In vitro* transcription and purification of tRNA^Sec^

All tRNA^Sec^ species were synthesized using *in vitro* T7 RNA polymerase run-off transcription. Briefly, the tRNA^Sec^ gene with a T7 RNA polymerase promoter directly upstream was amplified by PCR from the pUC19 vector using the following primers.

For amplification of all tRNA^Sec^ constructs, the following common *forward* primer was used:

Forward: 5′-CCCAGTCACGACGTTGTAAAACG -3

Along with one of the following species-specific *reverse* primers:

Human tRNA^Sec^: 5′-TGGCGCCCGAAAGGTGGAAT-3′

MMP tRNA^Sec^: 5′-GGCGGCGCAGGGGGGGAAT-3′

*E. coli* tRNA^Sec^: 5′-TGGCGGAAGATCACAGGAGTC-3′

After verifying the template sequence by Sanger sequencing, the transcription reaction was performed at +37°C for 4–5 h in 40 mM Tris, pH 8.0, 22 mM MgCl_2_, 2 mM spermidine, 0.01% (v/v) Triton X-100, 50 mg/ml PEG 8000, 50 μg/ml BSA, 10 mM DTT, 10 mM GMP, 4 mM of each rNTP (ATP, GTP, CTP, and UTP), 62.5–70 μl/ml of the tRNA^Sec^ PCR product (unpurified), and 50 μg/ml of T7 RNA polymerase. Prior to purification, precipitated magnesium pyrophosphate was pelleted by centrifugation and removed. The cleared reaction was loaded onto a Resource-Q column (Cytiva), and human and MMP tRNA^Sec^ were purified using a linear gradient of NaCl (0.4–0.7 M) in 20 mM Tris, pH 8.0, while *E. coli* tRNA^Sec^ purification used a 0.2–1 M NaCl gradient. Following elution, tRNA was further purified on a S75 16/60 Superdex SEC column (Cytiva) equilibrated in 20 mM HEPES, pH 8.0 and 150 mM NaCl. The eluted tRNA^Sec^ was concentrated to ∼100 μM, filtered through a 0.22 μm filter, flash-frozen in liquid nitrogen, and stored at −80°C.

### Thermal shift assay

Protein stability and integrity of WT and Δ470 SEPSECS were evaluated by comparing the changes in intrinsic fluorescence in thermal unfolding profiles from a Tycho instrument (NanoTemper Technologies). For sample preparation, the human enzymes were diluted to 1 mg/ml (∼17 μM) in 20 mM HEPES, pH 8.0, 150 mM NaCl, 5% (v/v) glycerol and 0.05% (v/v) Tween-20. MMP SepSecS lacks Trp residues resulting in much weaker intrinsic fluorescence, which consequently required a higher protein concentration to detect the changes in intrinsic fluorescence. To minimize the influence of any buffer alterations, MMP SepSecS was diluted to 5 mg/ml and human SEPSECS diluted to 1 mg/ml in the MMP protein storage buffer: 20 mM Tris, pH 8.0, 200 mM NaCl, 5% (v/v) glycerol, 10 μM PLP and 0.5 TCEP. The diluted protein samples were spun for 5 min at 12 000 rpm to pellet and remove any aggregated protein. Finally, samples were loaded into Tycho capillaries (NanoTemper Technologies) and analyzed in duplicate (or quadruplicate for MMP SepSecS).

### Electrophoretic mobility shift assay (EMSA)

For the assay, 4%, 0.5× TBE (45 mM Tris, 45 mM boric acid, 1 mM EDTA) polyacrylamide gels were cast using 1.5 mm spacer plates. For the tRNA^Sec^ titrations, each sample was prepared using 4 μg of WT or Δ470 SEPSECS with an increasing molar ratio of tRNA^Sec^ (i.e. 4:0, 4:1, 4:2, 4:4, 4:6 or 4:8). The appropriate amounts of protein and tRNA were then mixed, adjusted to 18 μl using 20 mM Tris (pH 8.0) and 150 mM NaCl, and allowed to equilibrate for 15 min at room temperature. For the tRNA^Sec^ sample, 0.5 μg of tRNA was similarly diluted to a volume of 18 μl. After equilibration, 2 μl of the 5× Hi Density TBE Buffer (Invitrogen) was added to the sample and mixed for a total volume of 20 μl, with 10 μl of each sample loaded onto the gel. For assessing SEPSECS binding to various tRNA^Sec^ species, 25 μl samples were similarly prepared in 20 mM Tris, pH 8.0 and 150 mM NaCl using 5 μg of MMP SepSecS or human SEPSECS mixed with a molar equivalent of each tRNA species: human tRNA^Sec^, human tRNA^Ser^, MMP tRNA^Sec^ or *E. coli* tRNA^Sec^. The gels were run in 0.5× TBE buffer at 100 V until the bromophenol blue dye front had migrated about ∼3/4 down the gel. To visualize tRNA bands, gels were stained in 50 ml of 0.5 μg/ml ethidium bromide (EtBr) in water for 10 min. To visualize protein bands, the same gel was subsequently stained with Coomassie Brilliant Blue R-250.

### Multi-angle light scattering (MALS) and refractive index (RI) measurements

SEC-MALS experiments were performed at BioCAT (Beamline 18-ID) at the Advanced Photon Source (Argonne National Laboratory). SEC-MALS experiments were conducted using an Agilent Infinity II HPLC (Agilent Technologies) coupled to a Wyatt DAWN HELEOS II MALS + DLS detector and a Wyatt Optilab T-rEX dRI detector (Wyatt Technology). MALS-RI measurements were taken on samples separated on a Superose 6 Increase 10/300 column (Cytiva) at a flow rate of 0.6 ml/min. Samples were prepared in 20 mM Tris, pH 8.0, 150 mM NaCl, 5% (v/v) glycerol, 0.5 mM TCEP, and 10 μM PLP. For protein alone, 250 μl of WT or Δ470 SEPSECS at 1 and 4 mg/ml were injected onto the column. tRNA^Sec^ was denatured by heating to 90°C for 2 min and renatured by allowing to cool to room temperature. For tRNA^Sec^ alone, 250 μl of 0.75 mg/ml human tRNA^Sec^ was used. For each titration series, SEPSECS was held constant at 1 mg/ml and tRNA was titrated in at a molar ratio of 1, 2, 4, 6 or 8 molecules per SEPSECS tetramer, with 250 μl of each sample injected onto the column. Prior to injection all samples were filtered through a 0.22 μm filter to remove any aggregated protein. The d*n*/d*c* value for all samples was defined as 0.185 ml/g for SEPSECS-containing samples and 0.172 ml/g for tRNA^Sec^. System equilibration was performed with 20 mM Tris, pH 8.0, 150 mM NaCl, 5% (v/v) glycerol, 0.5 mM TCEP and 10 μM PLP, and the system was normalized using bovine serum albumin according to the manufacturer's protocol. Data collection and analysis were performed with ASTRA 8.0 software (Wyatt Technology).

### Negative staining

Complexes of WT human SEPSECS or the truncated mutant Δ470 SEPSECS were prepared in the protein storage buffer (20 mM HEPES, pH 8.0, 200 mM NaCl, 5% (v/v) glycerol, 10 μM PLP and 0.5 mM TCEP). Prior to complex formation, tRNA^Sec^ was denatured by heating to 90°C for 2 min and renatured by cooling to room temperature. The protein was diluted to 0.04 mg/ml and mixed with an appropriate volume of tRNA^Sec^ to form complexes with a molar ratio of protein-to-tRNA at 4:1, 4:2 or 4:4. Copper 200 mesh grids with a carbon film (EMS Hatfield, PA) were glow discharged (Solarus, Gatan Pleasanton, CA) for 30 s and incubated with the pre-formed complexes for 60 s. The complex was blotted off, rinsed two times with 0.75% uranyl formate (EMS), and then stained with 0.75% uranyl formate for 45 s. Grids were imaged with a Tecnai G2 F30 electron microscope (FEI Hillsboro, OR).

### Comparative genomic analysis

Sequences orthologous to *Homo sapiens* SEPSECS were identified using a profile hidden Markov model homology search of both the Swiss-Prot and TrEMBL databases implemented in the phmmer algorithm of HMMER v3.1b2 (41) with the following parameters: (-E 1 –domE 1 –incE 0.001 –incdomE 0.03 –mx BLOSUM90 –pextend 0.4 –popen 0.02). Search results were filtered based on the criteria of protein length and alignment length to the query with a minimum of 300 amino acids and a maximum of 600 amino acids required for each. The homology search resulted in the identification of 874 orthologous protein sequences, 14 of which are archaeal with the remainder representing species across the breadth of eukarya. Multiple protein sequence alignment of these orthologs was conducted with kalign (42) using the full-length sequence, N-terminal, and C-terminal subsets for all species; and subset alignments of each of our taxonomic groups (amphibians, archaea, birds, fishes, fungi, invertebrates, mammals, protozoans, reptiles). Maximum-likelihood phylogenies were reconstructed with PhyML 3.0 using automatic substitution model selection and approximate likelihood-based branch supports (43–45). C-terminus origination in SEPSECS orthologs was inferred through inspection of the presence or absence of aligned C-terminal sequences, amino acid composition of any aligned C-terminal regions, and their placement on the 874 species-based phylogeny. Conservation scores for specific residues were determined using SnapGene, while pairwise identity was assessed using Geneious Prime 2024.0.7.

### Structural comparisons

The electrostatic potential surface for holo human and MMP SepSecS were calculated in PyMOL (version 2.4.2) with continuum electrostatic calculations using the Adaptive Poisson–Boltzmann Solver (APBS) software package plugin (46). The holoenzymes were converted to a PQR file using PDB2PQR. The PQR file was then analyzed by APBS using the default settings with a solvent probe radius of 1.4 Å, surface sphere density of 10 grid points/Å^2^. Temperature was set to 310 K, ionic strength to 0.15 M in monovalent salt, and the dielectric constants for solute (protein and ligands) and solvent to 2.0 and 78.00, respectively.

Secondary structure prediction for the 874 orthologous SEPSECS sequences was generated using S4PRED (47). To generate the structural models of vertebrate SEPSECS, we used the AlphaFold2-mmseqs2 notebook within ColabFold (48). For notebook settings, default parameters were used except the template mode was set to PDB100, the number of recycles to 12, and the recycle early stop tolerance was set to 0.0. Only the top-ranked structure from each ColabFold output was considered in our structural comparisons to the human holoenzyme. While ColabFold could generate the tetramer, only the monomeric structure was predicted to minimize the computational time. The predicted monomer was then individually aligned to each monomer of the holoenzyme to build the tetrameric structure. Evolutionary conservation scores were calculated and mapped onto the structure of the human SEPSECS holoenzyme using the ConSurf Web Server, which was run in the PDB_MSA_Tree mode with the Bayesian method, using PDBID 7L1T and Chain A along with our user-generated multi-sequence alignment and phylogenetic tree files (49–51). The SEPSECS tetramer was then assembled in PyMOL using the output Consurf-modified PDB file. All figures were made using PyMOL Molecular Graphics System, Version 2.4.2 Schrödinger, LLC.

### *E. coli* SepSecS complementation assay

The activity of human SEPSECS variants and MMP SepSecS were assessed by evaluating their ability to rescue the loss of SelA in Δ*selA* JS2(DE3) cells via the activity of the selenoenzyme, formate dehydrogenase (FDH) (40). The day prior to the assay, we inoculated LB broth with 1% (w/v) glucose, carbenicillin (100 μg/ml) and chloramphenicol (34 μg/ml) and grew each strain aerobically for 16 h at +37°C. Cells were centrifuged, resuspended, and diluted in sterile 1× PBS to a cell density of 4 × 10^9^ cells/ml. Each strain was serially diluted to a cell density of 4 × 10^5^ cells/ml. Subsequently, 10 μl of each dilution series was plated onto a row of square LB agar plates containing carbenicillin (100 μg/ml), chloramphenicol (34 μg/ml), 10 μM IPTG, 1 μM Na_2_MoO_4_, 1 μM Na_2_SeO_3_, 50 mM HCOONa and 0.5% (w/v) glucose, in duplicate. Plates were incubated in an anaerobic chamber (Type A vinyl 110V, Coy Lab Products) with a gas mix of 90% N_2_, 5% H_2_, 5% CO_2_ for 24 h at +25°C. The next day, the LB top agar (0.75% (w/v) agar) was prepared and supplemented with 1 mg/ml benzyl viologen (BV), 250 mM HCOONa and 25 mM KH_2_PO_4_ (pH 7.0). For each assay plate, 10 ml of the supplemented top agar was poured on and gently distributed to cover the plate. To visualize the BV reduction, plates were imaged 30 min after the overlay with the top agar. All experiments were performed in duplicate.

## Results

### α16 employs acidic surfaces to preclude electrostatic interactions between the SEPSECS non-catalytic protomer and tRNA^Sec^

We previously reported (34) that higher diffracting crystals of the human SEPSECS•tRNA^Sec^ complex (PDBID 7MDL and 8G9Z) yielded more informative *F*_o_− *F*_c_ electron density difference maps that revealed an alpha helical density near α14 of the non-catalytic, tRNA-free, protomer. Moreover, the α-helix featured extended side chain densities reaching out towards the side chain of Arg398 in the tRNA-free protomer, suggesting the presence of acidic residues in those positions. Since the extreme C-terminus harbors many acidic amino acids that could interact with Arg398, we modeled this new helix as α16, encompassing residues from Glu477 to Leu493. In the catalytic protomer, Arg398 is pivotal for establishing tRNA^Sec^ identity through interactions with the G73 discriminator base of tRNA^Sec^. Conversely, in the non-catalytic protomer, the side chains of Glu482 and Asp489 fully occupy the guanidinium group of Arg398 and prevent interactions with the discriminator base. Moreover, the placement of α16 sterically occludes the acceptor-TΨC arms from binding to the enzyme and approaching the active site crevice. Consequently, this change in α16 breaks the equivalency of these sites within the tetramer, leading to the observed structural asymmetry of the binary complex with a catalytic and non-catalytic protomer.

**Figure 1.**
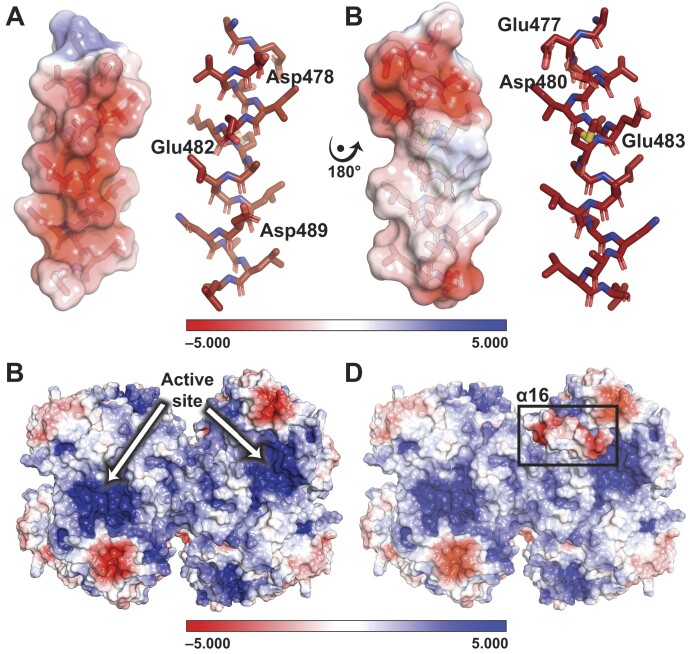
Helix α16 of human SEPSECS utilizes negatively charged surfaces to engage the tRNA^Sec^ binding pocket and entrance into the active site. Electrostatic potential maps of the α16 faces (PDBID 7MDL) show acidic residues along α16 comprise a negatively charged (red surfaces) face (**A**) and a more neutral face when rotated 180° along the y-axis (**B**). (**C**) Human holo SEPSECS (PDBID 7L1T) harbors large positively charged surfaces (blue) that constitute the tRNA binding pockets and active sites. (**D**) α16 nestles into the tRNA binding pocket of SEPSECS in a manner that orients the acidic face of α16 toward the tRNA binding pocket. As illustrated by the bar, surfaces are contoured from −5 (red) to +5 (blue) kT/e^−^ based on the potential of the solvent accessible surface.

A closer examination showed that α16 possesses a plethora of acidic residues. Plotting the electrostatic potential of α16 revealed that the arrangement of Asp478, Glu482 and Asp489 on one helical face comprises an acidic face (Figure 1A). Rotating α16 by 180° revealed a more neutral face of the helix with an acidic N-terminus comprised of Glu477, Asp480 and Glu483 (Figure 1B). As previously noted, the tRNA binding interface and catalytic pockets of human SEPSECS feature positively charged surfaces to facilitate engaging the negatively charged reaction substrates (Figure 1C) (34). tRNA binding causes the acidic face of α16 to nestle into a crevice of the basic tRNA binding pocket (Figure 1D). In this way, the acidic face of α16 engages residues critical for the recognition, binding, and catalytic activity of SEPSECS (34). Given the importance of charged interactions in mediating binding between SEPSECS and tRNA^Sec^, the composition and configuration of α16 appear designed to disrupt tRNA binding to the non-catalytic protomer. Such an arrangement suggests that α16 plays a key role in regulating the overall stoichiometry of SEPSECS at 4:2 and may help explain why, unlike bacterial SelA, SEPSECS is unable to bind the full complement of tRNA molecules, despite having four equivalent binding and catalytic sites.

### Truncation of the C-terminal tail does not alter the core architecture of human SEPSECS

To probe the functional relevance of the C-terminus, we inserted an early stop codon at the position encoding Asp470 (Δ470 SEPSECS) to eliminate the α15–α16 loop and α16. Earlier studies have shown that the catalytic core of mammalian SEPSECS comprises residues ∼19–468 (32,29). Thus, we expected that Δ470 SEPSECS would fold like the WT enzyme with all the elements necessary for substrate binding and catalysis. Indeed, MALS experiments confirmed that the truncated mutant still organizes as a tetramer (Table 1). Furthermore, WT and Δ470 SEPSECS showed comparable thermal unfolding profiles (Supplementary Figure S1) and inflection temperatures (Table 2), implying that Δ470 SEPSECS folds like the WT enzyme and that the C-terminal appendix is not part of the core structure.

**Table 1.** MALS-derived molecular weights of SEPSECS species

Sample	Theoretical MW^a^ (kDa)	Peak 1 MW (kDa ± %uncertainty)
**WT SEPSECS (1 mg/ml)**	232	210 (± 4.2%)
**WT SEPSECS (4 mg/ml)**	232	211 (± 3.6%)
**Δ470 SEPSECS (1 mg/ml)**	217	195 (± 3.0%)
**Δ470 SEPSECS (4 mg/ml)**	217	195 (± 2.7%)
**tRNA^Sec^ (0.75 mg/ml)**	29	29 (± 4.8%)

^a^Protein MW was calculated using the amino acid sequence and ProtParam, while the tRNA MW was calculated from the nucleotide sequence using IDT’s OligoAnalyzer.

**Table 2.** Thermal unfolding parameters for Δ470 SEPSECS

Sample	*T*_i_^a^	Initial ratio^b^	ΔRatio^c^
**WT**	74.1 ± 0.1	0.549 ± 0.001	0.247 ± 0.002
**Δ470**	74.6 ± 0.1	0.557 ± 0.001	0.232 ± 0.002

^a^ *T*_i_ is the inflection temperature of the unfolding transition in the signal of the 350 nm/330 nm ratio.

^b^Initial ratio is the value of the ratio of 350 nm/330 nm at the beginning of the measurement.

^c^ΔRatio is the difference between the ratio at the beginning and at the end of the thermal profile.

Both MALS and thermal unfolding showed that truncation did not alter the aggregation or oligomerization status of the holoenzyme. To rule out concentration-dependent effects, we characterized holo WT and Δ470 SEPSECS at 1 and 4 mg/ml. Each experiment generated a single peak corresponding to a monodisperse species with homogenous molar mass distributions at both the higher and lower concentrations (Supplementary Figure S2). The scattering signal intensity correlated with rising sample concentrations, but both concentrations generated a tetrameric molecular weight of ∼210 kDa for the WT and ∼195 kDa for Δ470 SEPSECS, eliminating the possibility of oligomerization. In addition, the polydispersity (*M*_w_/*M*_n_) of each holoenzyme was ∼1.000, indicating the species were devoid of aggregates and ruling out concentration effects. Similar values for the initial and Δratios between WT and Δ470 SEPSECS in the thermal unfolding analysis further indicated the truncated mutant is thermally stable and not more aggregation prone than the WT enzyme (Table 2). Altogether, these data establish that Δ470 SEPSECS is stable and similar in structure to the WT enzyme.

### At low tRNA^Sec^ concentrations Δ470 SEPSECS forms a higher-order binary complex

**Figure 2.**
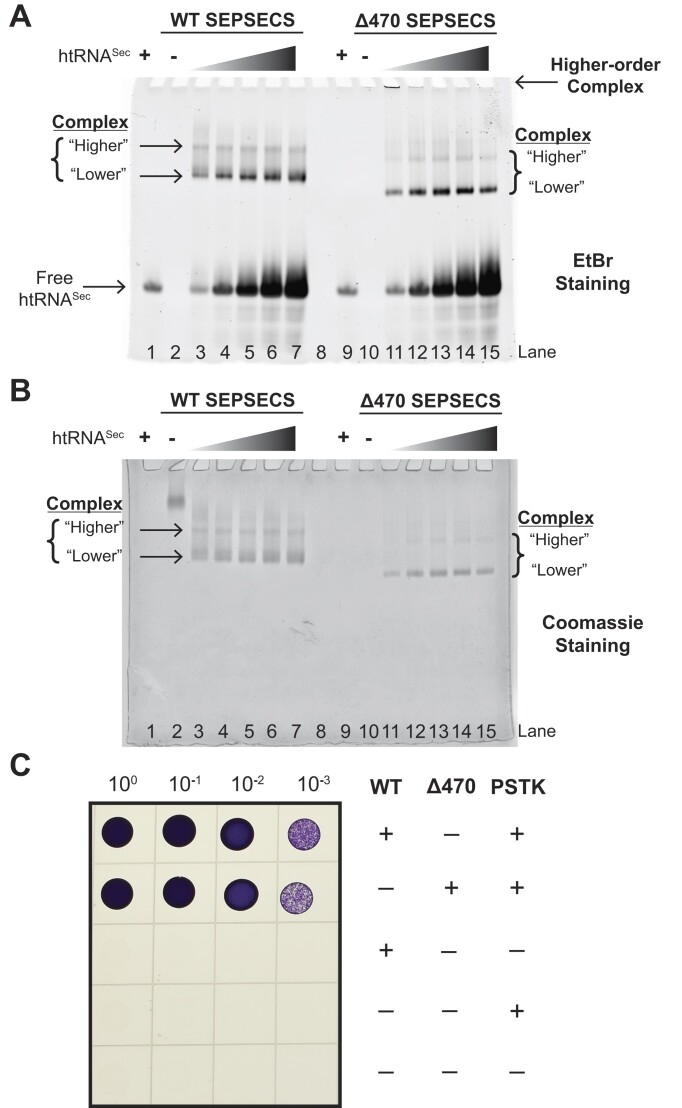
Δ470 SEPSECS maintains catalytic competency and forms higher-orders species at low concentrations of human tRNA^Sec^. (**A**) EtBr staining shows the change in migration of human tRNA^Sec^ (htRNA^Sec^) in a titration series upon binding to either WT or Δ470 SEPSECS. Binding of htRNA^Sec^ to WT or Δ470 SEPSECS formed soluble complex, as indicated by the ‘higher’ and ‘lower’ EtBr-staining bands with reduced electrophoretic mobility. However, binding to Δ470 SEPSECS induced a higher-order complex at low substrate concentrations, as indicated by staining within the well. The higher-order species was more prevalent with only one tRNA per tetramer (lane 11) and only minimally present when 2 tRNAs were provided per tetramer (Lane 12). (**B**) Coomassie staining of the same gel confirms the presence of protein in the complex. lane 1—free tRNA^Sec^, lane 2—WT, lane 3—4:1 WT: tRNA^Sec^, lane 4—4:2 WT: tRNA^Sec^, lane 5—4:4 WT: tRNA^Sec^, lane 6—4:6 WT: tRNA^Sec^, lane 7—4:8 WT: tRNA^Sec^, lane 8—empty, lane 9—free tRNA^Sec^, lane 10—Δ470, lane 11—4:1 Δ470: tRNA^Sec^, lane 12—4:2 Δ470: tRNA^Sec^, lane 13—4:4 Δ470: tRNA^Sec^, lane 14—4:6 Δ470: tRNA^Sec^, lane 15—4:8 Δ470: tRNA^Sec^. (**C**) Only co-expression of human SEPSECS and *Methanocaldococcus jannaschii* PSTK allows Δ*selA* JS2(DE3) cells to synthesize formate dehydrogenase and reduce benzyl viologen (BV) to its purple form. Δ470 SEPSECS exhibits similar levels of BV reduction as the WT enzyme throughout the dilution series. Experiment was performed in duplicate with one representative result shown.

Since α16 docks within the tRNA-binding motif of the non-catalytic protomer, we postulated that this α-helix might govern the overall stoichiometry of the binary complex. Therefore, we performed an EMSA to examine how deletion of α16 would impact the number of tRNA^Sec^ molecules binding to Δ470 SEPSECS (Figure 2). Binding of human tRNA^Sec^ to the WT enzyme generated two complex bands: a prominent, ‘lower’ band and a secondary minor species showing as a higher band in the gel (Figure 2A, B, lanes 3–7). We speculated that these bands represent complexes with one (higher) or two (lower) tRNA^Sec^ molecules bound, since increasing the mass of the nucleic acid component generally increases the relative mobility of a protein-nucleic acid complex (52,53). The WT complex exhibited fainter bands at a 4:1 protein-to-tRNA ratio that appeared saturated at a 4:2 ratio. This saturation is consistent with previous work that established the half-sites occupancy of tRNA binding sites where up to only two tRNA molecules bind the tetramer (33).

The truncated mutant displayed a similar pattern of two complex bands that migrated slightly further down the gel compared to their WT counterparts, with complex binding also saturated at a 4:2 protein-to-tRNA ratio (Figure 2A, B, lanes 11–15). Given that the complex appeared saturated at the same 4:2 molar ratio, it seemed unlikely the mutant bound a different number of tRNA molecules within this assay. The observed differences in migration between the WT enzyme and the mutant are likely attributable to distinct charge-to-mass ratios for each. Whereas the WT enzyme has an isoelectric point (pI) of 8.3 and was largely immobile in the EMSA buffer system (Figure 2B, lane 2), the Δ470 mutant, with a calculated pI of 9.2, migrated toward the cathode during electrophoresis (Figure 2B, lane 10). The Δ470 mutant truncates residues 471–501, a region with a pI of 3.31 due to 10 acidic amino acids (seven Asp and three Glu), which would yield an enzyme with a higher pI than the WT enzyme.

Unexpectedly, we observed a higher-order species of the binary complex for the Δ470 SEPSECS samples at lower tRNA concentrations (≤4:2). The negatively charged and soluble tRNA^Sec^ should easily migrate into the gel and towards the positive electrode. However, EtBr staining within the wells of the Δ470 SEPSECS complexes at molar ratios of 4:1 and 4:2 (Figure 2A, lanes 11–12) suggested that low tRNA concentrations induced formation of a higher-order protein-nucleic acid complex. Notably, this effect appeared to be dose-dependent, as lower tRNA concentrations enhanced formation of the higher-order species. However, with an EMSA it is difficult to discern whether these are higher-order oligomers that are too large to enter the gel or an aggregated species.

Despite these findings, deletion of the C-terminal tail exhibited minimal impact on catalytic activity in an *E. coli* complementation assay (Figure 2C). This functional assay relies on expression of active archaeal PSTK and human SEPSECS to compensate for the loss of bacterial SelA in bacterial Sec synthesis. Additionally, the assay is dependent on SEPSECS binding and acting on btRNA^Sec^ rather than human tRNA^Sec^. Yet, Δ470 SEPSECS binding to btRNA^Sec^ displayed an increased tendency to form a higher-order species *in vitro*, and this behavior persisted across the titration series with no soluble complex evident at the lowest titration point (Supplementary Figure S3). Despite these disparities in tRNA binding, Δ470 SEPSECS exhibited comparable levels of BV reduction as the WT enzyme across the dilution series (Figure 2C). The relevance of the higher-order species within an *in vivo* setting remains unclear. Taken together, α16 appears to regulate tRNA binding to ensure the overall stability of the complex.

### The Δ470 SEPSECS•tRNA^Sec^ binary complex aggregates at low tRNA^Sec^ concentrations

**Figure 3.**
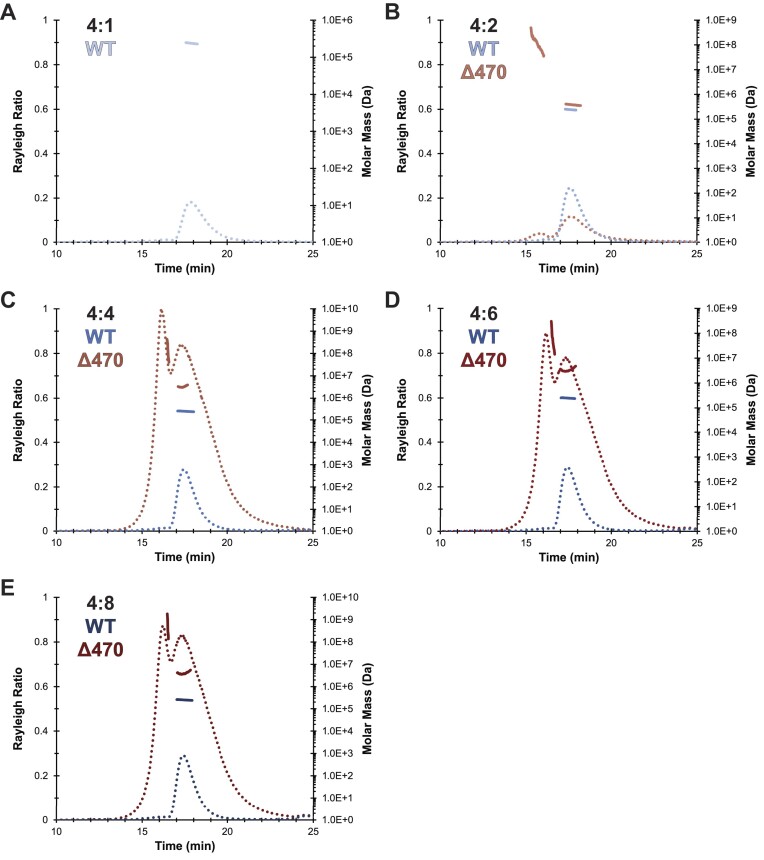
MALS traces reveal large, polydisperse species of the Δ470 SEPSECS binary complex. Light scattering traces of WT SEPSECS (blue) reveal a single, monodisperse species with a horizontal molar mass distribution across the peak that corresponds to the SEPSECS•tRNA^Sec^ binary complex. Analogous Δ470 SEPSECS samples (red traces) possess an aggregate peak (earlier eluting peak), and polydispersity within the sample precludes accurate molecular weight determination of the Δ470 SEPSECS species. MALS traces at SEPSECS•tRNA^Sec^ molar ratios of (**A**) 4:1, (**B**) 4:2, (**C**) 4:4, (**D**) 4:6, (**E**) 4:8.

To characterize the nature of the higher order Δ470 SEPSECS•tRNA^Sec^ complexes, we performed MALS to determine their molar mass. Addition of 1, 2, 4, 6 or 8 tRNA^Sec^ molecules per WT SEPSECS tetramer generated light scattering traces for a monodisperse species with a molar mass ranging from ∼235 to 250 kDa (Figure 3, Supplementary Table S1), consistent with SEPSECS binding one or two tRNA^Sec^ molecules. As expected, a higher molar ratio of tRNA^Sec^ increased the likelihood for two tRNA molecules being bound (Supplementary Table S1). Saturation of the tetramer occurred with a molar ratio of 4:6, with no further shifts of the scattering curve or corresponding increases in the molecular weight determination.

Conversely, Δ470 SEPSECS complex formation exhibited an abnormal phenomenon. Addition of one or two molar equivalents of tRNA^Sec^ resulted in immediate and visible aggregation of the complex, with the 4:1 titration point lacking any discernible signal for tRNA^Sec^ at 260 nm or Δ470 SEPSECS at 280 nm (Figure 3A). However, the addition of 4-, 6- or 8-fold molar equivalents of tRNA^Sec^ did not cause visible precipitation. Notably, the light scattering traces for all Δ470 SEPSECS complexes consistently exhibited two polydisperse peaks (Figure 3B–E). The later peak was readily observable in samples with ≥2 tRNAs per SEPSECS tetramer and aligned with elution of the WT complex, suggesting it largely corresponded to a stably assembled binary complex. In contrast, the earlier peak represented an aggregate peak with a high polydispersity and a vertical molar mass distribution. The irregular molar mass distribution across both Δ470 SEPSECS•tRNA^Sec^ peaks confirmed that tRNA binding induces aggregation rather than oligomerization and indicated that these aggregates are quite large. Since larger particles scatter light more strongly than smaller particles, the increased intensity of the scattering signal (and hence the Rayleigh ratio) is consistent with larger particles present in both species. Unfortunately, the sensitivity of MALS to even minor high-MW aggregates and the resulting polydispersity made it impossible to accurately determine the molecular weight (Figure 3). Thus, we were unable to determine if the C-terminal tail regulates the number of bound tRNA molecules.

Lastly, we performed negative staining electron microscopy experiments, which corroborated the dose-dependent aggregation behavior of Δ470 SEPSECS. WT SEPSECS formed stable complexes across the titration range (Supplementary Figure S4A, C, E) with minimal precipitation of the uranyl formate stain. Conversely, Δ470 SEPSECS exhibited widespread clumping of the stain with only one molar equivalent of tRNA^Sec^, indicating large-scale aggregation of the complex (Supplementary Figure S4B). Higher magnification showed a lower level of tetrameric species present compared to the WT sample, indicating that a significant amount of sample had precipitated. However, with a molar ratio of ≥2 tRNAs per tetramer, the level of uranyl formate precipitation and number and shape of tetrameric species resembled the WT complexes (Supplementary Figure S4D, F).

Altogether, these data describe an unusual binding phenomenon whereby the absence of the C-terminal tail renders tRNA binding unstable at sub-saturating molar ratios of tRNA^Sec^ (≤4:2), inducing complex aggregation. Aggregation is more prevalent at lower tRNA^Sec^ concentrations, while at higher molar ratios Δ470 SEPSECS•tRNA^Sec^ resembles the WT complex. Neither the truncated protein nor tRNA^Sec^ was unstable or aggregation prone (Table 2, Supplementary Figure S2), implying that Δ470 SEPSECS with only one tRNA molecule bound is destabilizing and aggregation prone. These findings further support the notion that the C-terminal tail of SEPSECS stabilizes the SEPSECS•tRNA^Sec^ complex.

### Archaeal SepSecS does not bind unacylated tRNA^Sec^

**Figure 4.**
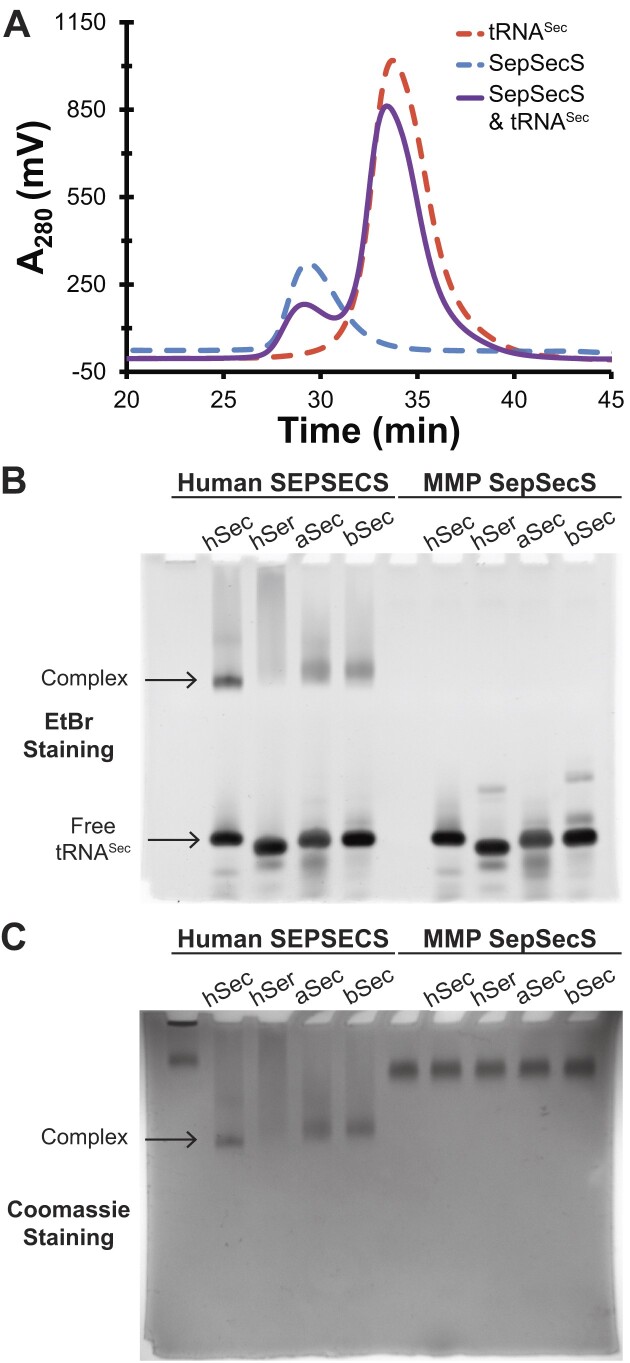
*M. maripaludis* SepSecS does not form a stable complex with unacylated tRNA^Sec^. (**A**) Size-exclusion chromatograms showed no observable binary complex peak upon mixing MMP SepSecS and MMP tRNA^Sec^. Instead, two peaks that coincide with the protein and MMP tRNA^Sec^ alone, respectively, are observed. The EMSA gel analyzing binding of human SEPSECS and archaeal SepSecS to several tRNA^Sec^ species, using either EtBr (**B**) or Coomassie staining (**C**). When mixed with a molar equivalent of tRNA, human SEPSECS formed a stable complex with human (hSec), MMP (aSec) and *E. coli* (bSec) tRNA^Sec^. The smear observed in the presence of human tRNA^Ser^ (hSer) indicated nonspecific binding. MMP SepSecS did not interact with any of the unacylated tRNA^Sec^ species.

Given the role of α16 in stabilizing complex formation of the human enzyme, we naturally wondered how archaeal SepSecS, which universally lack a C-terminal tail (32), could effectively bind tRNA^Sec^. We sought to delineate MMP SepSecS-tRNA^Sec^ binding in a manner analogous to the human enzyme. Unfortunately, attempts at complex formation yielded no discernible complex (Figure 4A), as SEC chromatograms exhibited no complex peak with a shorter retention time. Rather, the MMP SepSecS and MMP-tRNA^Sec^ mixture maintained two distinct peaks that aligned with the retention times of the free enzyme and free tRNA, respectively.

To further corroborate the absence of binding to unacylated tRNA^Sec^, we assessed the ability of MMP SepSecS to bind other species of tRNA^Sec^ using an EMSA. The human enzyme bound tRNA^Sec^ from various species, including *E. coli* tRNA^Sec^, requiring only the longer acceptor-TΨC helix to permit binding (Figure 4B, C). The smear observed with the shorter 7/5 acceptor-TΨC helix present in tRNA^Ser^ indicated that SEPSECS could only nonspecifically interact with the tRNA, consistent with previous binding data showing that a 7/5-tRNA^Sec^ mutant nonspecifically bound human SEPSECS (34). Strikingly, archaeal SepSecS was unable to bind its own tRNA^Sec^ nor any of the other species of tRNA^Sec^. The absence of binding was not due to enzyme and/or tRNA instability. Human SEPSECS binding to unacylated MMP-tRNA^Sec^ validated that MMP-tRNA^Sec^ was correctly folded. The thermal unfolding profile of MMP SepSecS indicated it was not aggregated or unfolded. Unlike the human enzyme, MMP SepSecS exhibited a bimodal unfolding profile, though only one inflection temperature (T_i_) was consistently distinct (Supplementary Figure S5). Regardless, the relatively high T_i_ values of ∼+70 °C indicated that the enzyme was not unfolded and was stable (Supplementary Table S2). Finally, the *E. coli* complementation assay verified that MMP SepSecS was catalytically competent when acting on an acylated tRNA substrate, Sep-btRNA^Sec^, since it enabled FDH synthesis to reduce the BV substrate (Supplementary Figure S6). This finding agrees with previous *in vitro* studies that demonstrated that MMP SepSecS catalyzes the substitution of the phosphoryl group when acting on aminoacylated MMP Sep-tRNA^Sec^ (30). Notably, while the MMP SepSecS strain could reduce BV, the lower intensity of BV reduction at the 10^−2^ dilution factor hinted it may be less efficient than the human enzyme.

In contrast to the human enzyme, MMP SepSecS failed to bind any unacylated tRNA^Sec^, presumably relying on the phosphoseryl group and binding to only aminoacylated tRNA^Sec^. However, such a dependency suggests the archaeal enzyme utilizes a distinct substrate binding mechanism, which could explain why the C-terminal tail is important for stabilizing tRNA binding in the human enzyme but is unnecessary in the archaeal enzyme.

### The tRNA binding interface of human SEPSECS is not strictly conserved

The EMSA revealed distinct electrophoretic mobilities for human SEPSECS and MMP SepSecS (Figure 4C). Archaeal SepSecS migrated farther towards the positive electrode, suggesting it possessed a lower pI and was more negatively charged. Conversely, human SEPSECS minimally migrated into the gel, consistent with a pI near the pH of the buffer system (∼8.3) (Figure 4C). Indeed, the theoretical pI, derived from the primary amino acid sequence of the His-tagged human SEPSECS or MMP SepSecS was 8.3 and 8.1, respectively. We hypothesized that a lower pI for MMP SepSecS could reflect an absence or neutralization of the Arg and Lys residues that facilitate stabilizing interactions with the negatively charged tRNA to allow complex formation.

Unfortunately, no structures of MMP SepSecS in complex with tRNA^Sec^ currently exist to directly delineate the interactions governing archaeal SepSecS complex formation. However, structural superimposition of the MMP SepSecS and human SEPSECS holoenzymes established that both enzymes share a similar tetrameric organization with a similar configuration of the active sites and putative binding helices: α1, α9, α14 and α15 (Supplementary Figures S7A). Whereas mouse and human SEPSECS essentially resembled one another (R.M.S.D. < 1 Å), the archaeal structure diverged more from the human enzyme (R.M.S.D. of ∼2 Å) (Supplementary Figures S7). Catalytically relevant amino acids in the PLP binding site and phosphate binding loop (P-loop) in the human and MMP enzymes were similarly positioned, implying a conserved catalytic mechanism (Supplementary Figure S8). Yet of the binding helices, only α9 adopts a similar helical axis as in the human enzyme (Supplementary Figure S9D–F), while helices α1, α14 and α15 exhibited offset orientations (Supplementary Figure S9A–C, G–O). Notably, α1 in MMP SepSecS possesses a plethora of acidic residues along its solvent-exposed face that would likely repel docking of the acceptor-TΨC helix of tRNA^Sec^ as occurs in human SEPSECS (Supplementary Figure S9A–C). α14 in MMP SepSecS also features more acidic residues and lacks a basic residue equivalent to Arg398 in human SEPSECS for recognizing the discriminator base (Supplementary Figure S9J). Instead at this position, MMP SepSecS harbors Leu384, which cannot hydrogen bond with G73 (Supplementary Figure S9K). Although MMP SepSecS has an Arg at position 385, its guanidinium group is distant and would clash with Leu384 to adopt an orientation like Arg398 in human SEPSECS. Thus, recognizing G73 using Arg385 in MMP SepSecS would necessitate the GCCA-end of tRNA^Sec^ to adopt a different orientation or angle of entry than in humans. Finally, α15 is shorter in MMP SepSecS (Supplementary Figure S9M-O) and thus lacks equivalent residues for Lys463 to help anchor the 5′-phosphate binding groove of tRNA^Sec^ as occurs in human SEPSECS (35). Overall, the structural data show that the helices critical for tRNA binding in the human enzyme are not conserved in MMP SepSecS.

**Figure 5.**
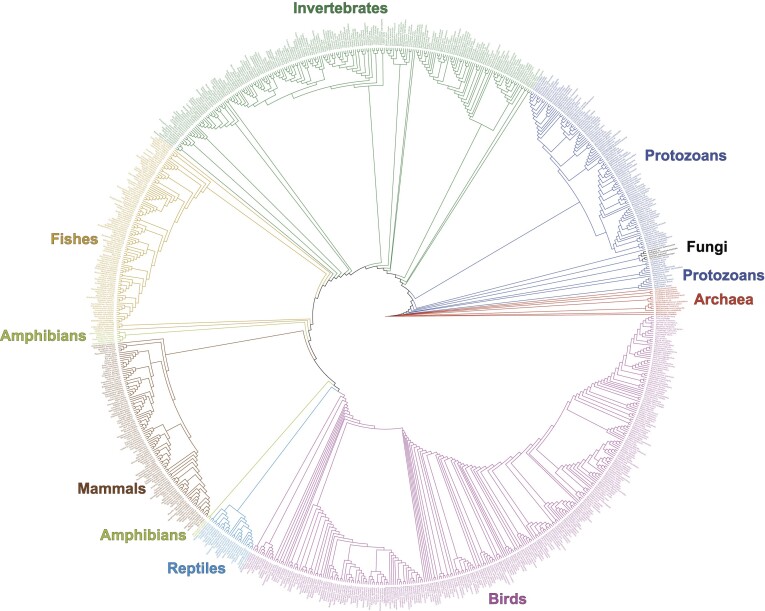
Phylogenetic reconstruction of SepSecS evolution. 874 SepSecS orthologs spanning from archaea to eukarya were used to reconstruct the evolutionary history of SepSecS, rooted at *M. kandleri*. Archaeal orthologs are colored in red, while the eukaryotic orthologs are divided into eight groups: protozoans (dark blue), fungi (black), invertebrates (dark green), fishes (yellow), amphibians (light green), mammals (brown), reptiles (light blue), and birds (pink). The cladogram illustrates the divergence of eukaryotic orthologs from archaeal orthologs, as well as the divergence of mammals from other vertebrates. An alternate view of the phylogeny with support values and branch lengths is available as online supporting materials in Supplementary Figure S14.

**Figure 6.**
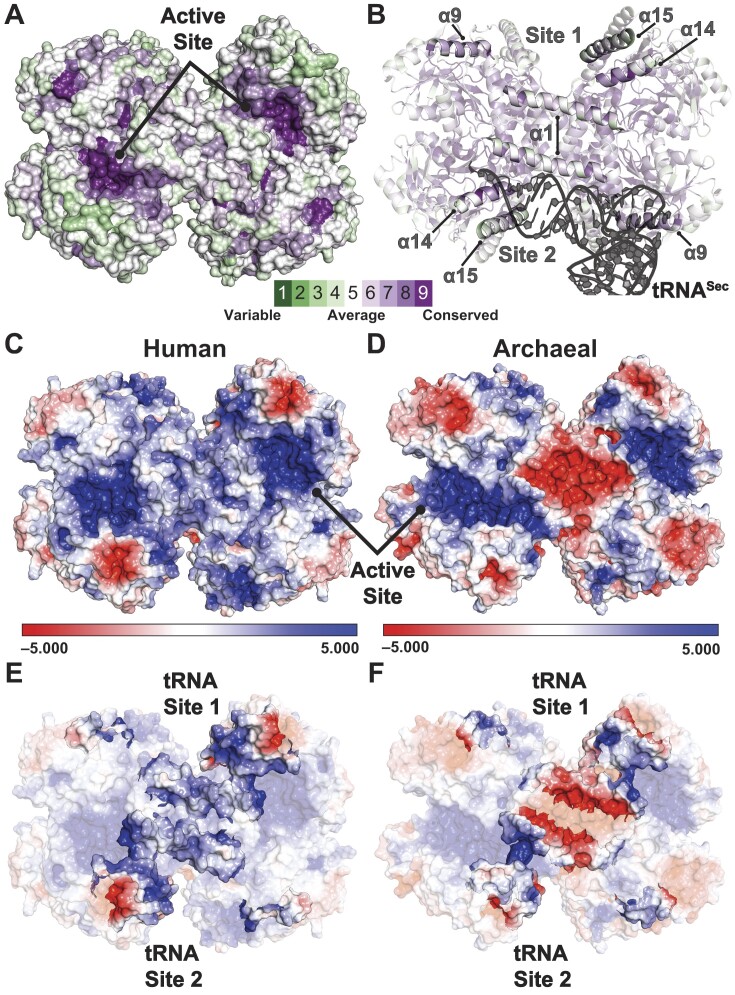
The enzyme surface including the tRNA-binding motifs are weakly conserved. (**A**) Consurf-generated evolutionary conservation grades mapped onto a surface representation of human holo SEPSECS (PDBID 7L1T). The active sites are strongly conserved (purple), while the enzyme surface has more variability (green). (**B**) Conservation of α1, α9, α14 and α15 which comprise two tRNA-binding pockets across each face of the human enzyme, with human tRNA^Sec^ (PDBID 7MDL) modeled onto Site 2. (**C**) The electrostatic potential map of human SEPSECS (PDBID 7L1T) largely features a positively charged solvent-exposed surface (blue). (**D**) Apart from the positively charged active sites, archaeal SepSecS (PDBID 2Z67) exhibits a more negatively charged surface (red), particularly across the center of the tetramer. (**E**) Helices α1, α9, α14 and α15 comprise the positively charged tRNA binding pocket (tRNA Site 1 and 2). (**F**) The comparable helices in archaeal SepSecS generate more acidic surfaces. As illustrated by the bar, surfaces are contoured from –5 (red) to +5 (blue) kT/e^−^ based on the potential of the solvent accessible surface.

To validate that the tRNA binding mechanism is not well-conserved and confirm that MMP SepSecS is representative of archaeal species, we conducted a phylogenetic analysis of SepSecS orthologs across archaea and eukarya. Consistent with previous studies, our phylogenetic reconstruction of 874 SepSecS orthologs showed a deep divide between the archaeal and eukaryotic versions of these enzymes (Figure 5 and Supplementary Figure S14) (30). MMP SepSecS shares a considerable degree of sequence similarity with the other methanogenic archaea considered in this study (Supplementary Figure S10). Notably, the phylogenetic tree indicated substantial changes to the enzyme occurred even within the eukaryotic lineage, particularly prior to the emergence of vertebrates. Across the analysis, the conservation of the catalytic centers (i.e. the P-loop) was markedly higher than for the helices comprising the tRNA binding pocket (Supplementary Figure S11). Catalytic residues exhibited higher conservation scores and % pairwise sequence identity (∼≥70%) compared to residues identified as having a role in binding (∼40–70%) (Supplementary Table S3). Mapping the ConSurf-generated conservation scores onto the structure of the human holoenzyme showed the strong conservation of the catalytic clefts and relatively weaker conservation of the enzyme surface and putative tRNA binding sites (Figure 6A, B and Supplementary Figure S12). The amino acids comprising α1 exhibited especially low conservation (Supplementary Figures S11A and S12A), suggesting that docking of tRNA^Sec^ by α1 is a more recently derived function. For instance, Lys38 in α1 of human SEPSECS helps bind tRNA^Sec^, and its absence substantially weakens binding affinity (34). Yet, early versions of the enzyme utilize a His or Gln at this position. Within vertebrates, mammals and birds generally use Lys, whereas the more basal vertebrate species of SEPSECS often use Gln. Additionally, archaeal SepSecS species typically harbor a conserved Leu at position 384 that cannot engage G73 (Supplementary Figure S9J–L). Leu is the conserved residue in the majority of archaeal species sampled in this work (11/14). Only two methanoarchaea and one species of Asgardarchaeota have either an Arg or Lys residue in this position, suggesting that the ability to recognize the discriminator base had not yet been fixed across archaea prior to eukaryotic divergence (Supplementary Figure S10). In contrast, Arg occurs in this position near the base of the eukaryotic lineage, emerging in the early protozoans, though it is occasionally substituted with a Lys or rarely a His, Ser, or Cys. Thus, employing an Arg to recognize the discriminator base is likely a general mechanism of tRNA recognition in eukaryotic SEPSECS. Overall, the phylogenetic analysis corroborated our structural comparisons, showing that the tRNA binding mechanism utilized by human SEPSECS evolved over time and was not present at the emergence of the enzyme.

Mapping the electrostatic potential onto the MMP SepSecS holoenzyme highlighted a more negatively charged surface than the human enzyme (Figure 6C, D), confirming findings from the EMSA and structural superimpositions. MMP SepSecS features a prominently acidic surface across the center of the enzyme and narrow, positively charged, catalytic clefts. Probing the purported binding interface showed that MMP SepSecS is less positively charged across α14 and is negatively charged across α1 (Figure 6E, F). To assess if an acidic surface is characteristic of archaeal SepSecS, we calculated the pI across the corresponding sequences of α1, α9, α14 and α15 from the other archaeal species. In mammalian SEPSECS, the pI across all these helices is basic (Table 3), consistent with the electrostatic potential surface maps (Figure 6E). However, basicity is only conserved in α9 among the archaeal species. The methanogenic archaea generally feature more negatively charged faces across the binding helices, especially helices α14 and α15. A more negative charge distribution across methanogenic archaeal SepSecS would likely preclude tRNA binding by the same mechanism as in humans. SepSecS orthologs from Asgardarchaeota, an archaeal phylum nearer to the prokaryote-eukaryote interface (54), more closely resemble the mammalian orthologs with more positively charged α14 and α15 helices. The charge across α1 is not conserved among archaea, fluctuating between acidic and basic. This variability is unsurprising as α1 appears to be the most recently acquired of the four binding helices (Supplementary Figures S11A and S12). Ultimately, the shift from acidic to basic solvent-exposed surfaces in SepSecS also helps explain the weaker conservation of the enzyme surface (Figure 6A).

**Table 3.** Theoretical pI values for alpha helices with a role in tRNA^Sec^ binding

Species	pI of α1	pI of α9	pI of α14	pI of α15
**Mammalian species**				
**Human**	9.19	9.52	11.39	10.18
**Mouse**	8.08	9.52	11.39	9.21
**Archaeal species**				
***Thorarchaeota archaeon***	11.60	10.64	8.99	10.58
***Candidatus Jordachaeia***	5.35	11.62	8.99	4.70
***Candidatus Helarchaeota***	8.99	10.64	8.99	9.02
***Candidatus Bathyarchaeota***	4.12	9.04	8.94	4.27
***Candidatus Prometheoarcha* *eum***	10.9	9.33	6.15	10.35
***Candidatus Lokiarchaeota***	10.64	9.18	3.62	4.27
***Lokiarchaeum* *sp***.	11.10	6.16	8.83	9.17
***Methanothermococcus okinawensis***	7.03	10.63	8.94	10.19
***Methanofervidicoccus abyssi***	9.17	10.62	4.03	4.11
***Methanotorris formicicus***	4.76	10.38	5.90	6.35
***Methanocaldococcus jannaschii***	10.18	10.16	3.84	4.62
***Methanococcus maripaludis***	5.51	10.16	4.03	6.27
***Methanopyri archaeon***	8.99	8.99	6.03	4.41
***Methanopyrus kandleri***	5.48	8.99	6.03	4.70

^a^pI values for each helix were calculated using Geneious Prime 2024.0.7.

Overall, the EMSA, structural comparisons, and phylogenetic analysis all demonstrated that human SEPSECS has evolved positively charged, solvent-exposed surfaces that allow for tRNA recognition and provide stabilizing interactions to favor complex formation. These data help clarify why archaeal SepSecS was unable to bind unacylated tRNA^Sec^.

### α16 in human SEPSECS is a mammalian innovation

**Figure 7.**
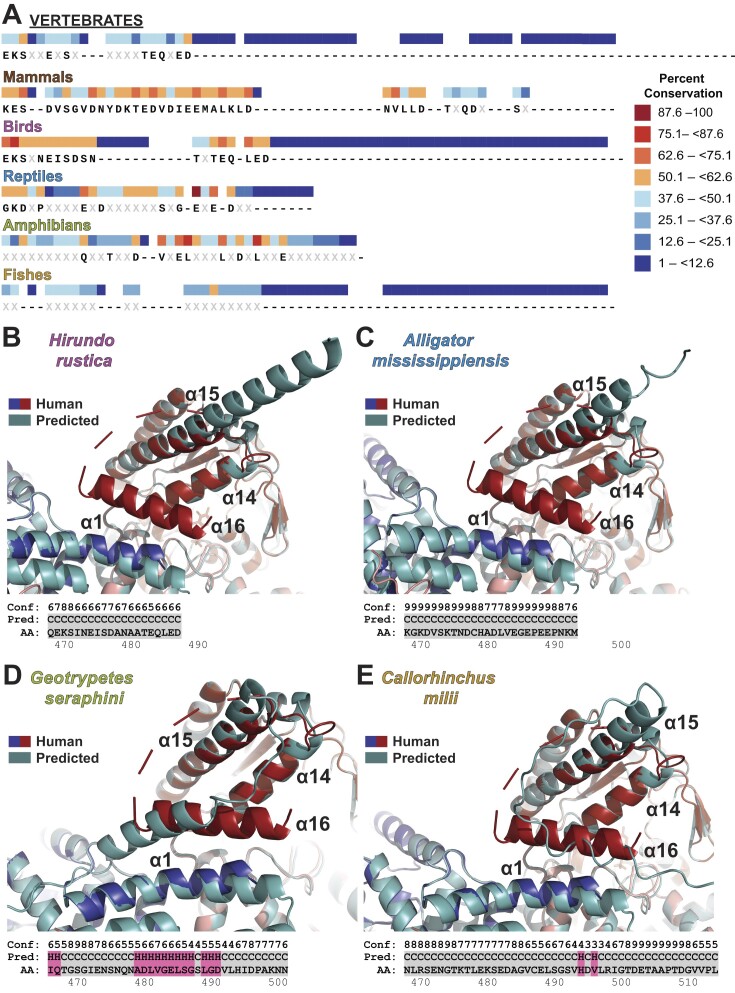
The vertebrate lineages have distinct amino acid sequences for the extended C-terminus of SEPSECS. (**A**) The extreme C-terminal region of SEPSECS shows poor conservation among vertebrates. Within vertebrates, mammals, birds, reptiles, and amphibians have unique consensus sequences (≥50% conservation). (**B–E**) Representative structural predictions for the extended C-terminus in non-mammalian, vertebrate clades, using ColabFold (top) and S4PRED (bottom). ColabFold structures (light teal cartoon) are shown aligned to the human SEPSECS (PDBID 7MDL), with the catalytic protomer colored blue and the non-catalytic protomer in red. The S4PRED predictions indicate a helical conformation (highlighted in pink) or a coiled conformation (highlighted in gray), with confidence scores noted above.

Consistent with previous studies, no archaeal species exhibited an extended C-terminus beyond α15. Prior to invertebrates, any C-terminal extensions only occurred sporadically and were not stably conserved. Within some of the invertebrate clades an extended C-terminus was present, though they were often rich in Gly and/or Pro residues and predicted to adopt coiled conformations. The divergence of vertebrates coincided with the emergence of an extended C-terminal sequence. However, the extended C-terminal sequences amongst vertebrates were not orthologous with no apparent consensus sequence (<50%) (Figure 7A). To investigate intraspecific patterns of sequence evolution, we conducted multiple sequence alignments for each vertebrate clade (mammals, birds, reptiles, amphibians and fishes) considered in this study. Mammals possess an acidic C-terminal sequence evident at the base of the mammalian branch with the divergence of *Ornithorhynchus anatinus*. Avian SEPSECS orthologs possess an acidic, yet significantly shorter C-terminal sequence compared to the other vertebrates. The reptilian C-terminal consensus sequence, while acidic, notably contains conserved helix-disrupting residues, Pro and Gly. Amphibian SEPSECS orthologs have several conserved acidic residues in the C-terminus, whereas fish do not exhibit conservation of this region. The poor interspecific sequence conservation of the vertebrate C-terminal extension, coupled with its restriction to vertebrates, suggests that it was not inherited from a common ancestor and emerged in separate evolutionary events.

Subsequently, we used S4PRED and ColabFold to determine if the extended C-terminus among the non-mammalian vertebrate species would also generate a helical conformation that could aid in orienting acidic residues to engage the tRNA binding pocket. The top ranked ColabFold structures for the representative, non-mammalian, vertebrate species of SEPSECS generally agreed with the S4PRED secondary structure prediction (Figure 7B–E). However, for *Hirundo rustica* (avian) SEPSECS, ColabFold predicted an extended α15, whereas S4PRED predicted a coiled conformation. In general, the quality scores of the ColabFold prediction in the far C-terminus are poor due to the lack of sequence coverage in that region (Supplementary Figure S13). Nonetheless, corroboration of ColabFold-predicted structures with S4PRED increased our confidence in the prediction of an α16. The shorter avian C-terminal sequence likely lacks the length to interact with the tRNA-binding site, regardless of whether it adopts an extended α15 as predicted by ColabFold or a coiled conformation as predicted by S4PRED. Among reptiles, amphibians, and fishes, some species have a predicted acidic α16, though it is not uniformly present (Figure 7B–E). Since the ColabFold structures of SEPSECS from non-mammalian vertebrates generally oriented α16 as in the human enzyme, it seems plausible that it may perform an analogous function in regulating tRNA binding. Thus, while there appears to have been some advantage to evolve an analogous helical and acidic C-terminal domain in vertebrate species of SEPSECS, it is unclear what that was.

## Discussion

Using biophysical, biochemical, and phylogenetic approaches, we aimed to determine the origin of the appended C-terminus in human SEPSECS and establish its role in defining the quaternary structure of the terminal Sec-synthetic complex. Although the C-terminus of SepSecS was crucial to the discovery of the enzyme, its function was largely ignored, perhaps because its absence from archaea and lower eukaryotes suggested little functional significance. Recent high-resolution crystal structures of the human SEPSECS•tRNA^Sec^ complex revealed that α16 contributes to the structural asymmetry of substrate binding by occluding the tRNA binding pockets of the neighboring monomers, thereby marking them as the non-catalytic protomer. Other PLP-dependent enzymes that act on a tRNA substrate, such as SelA and SepCysS, do not exhibit such half-sites occupancy.

Our findings demonstrate that the newly discovered α16 in human SEPSECS stabilizes the SEPSECS•tRNA^Sec^ binary complex. Deletion of the C-terminal tail minimally impacts the overall architecture of SEPSECS but significantly distorts tRNA binding, resulting in aggregation of the Δ470 SEPSECS•tRNA^Sec^ binary complex. Notably, this aggregation occurred in a dose-dependent manner with lower tRNA concentrations exacerbating aggregation, suggesting that Δ470 SEPSECS•tRNA^Sec^ with a single tRNA bound is unstable. Aggregation may directly result from the lower stability of a complex of Δ470 SEPSECS with a single tRNA. Alternatively, given the large positive surface on human SEPSECS and its ability to nonspecifically interact with human tRNA^Ser^, the α16 conformational change may prevent the open sites on the unbound face from nonspecifically or incorrectly engaging tRNA^Sec^. In this scenario, aggregation could result if a Δ470 SEPSECS tetramer bound to a single tRNA uses its unbound face to incorrectly engage tRNA^Sec^ already complexed with another tetramer. Irregular and non-productive binding could lead to aggregation and precipitation of Δ470 SEPSECS•tRNA^Sec^ complexes. Higher molar ratios of tRNA would then minimize aggregation since fewer sites would be available for incorrect binding. In the WT enzyme, binding of a single tRNA induces the α16 conformational change, partially blocking the basic surface of the unbound face, which would preclude an aggregation-inducing binding mode. Unfortunately, the irregularity of aggregation made it difficult to assess whether the deletion of α16 impacted the affinity or stoichiometry of binding. Mutational analysis of the acidic amino acids or helix disruption in α16 might better assess these parameters.

Deletion of the C-terminal tail did not noticeably alter the catalytic activity of SEPSECS in the *E. coli* complementation assay, despite the increased aggregation propensity of the Δ470 SEPSECS•btRNA^Sec^ binary complex. However, this assay best assesses catalytic competency and is not an accurate physiological reporter for human SEPSECS function and regulation. Firstly, overexpression of SEPSECS and PSTK could overcome enzyme loss due to aggregation with *E. coli* tRNA^Sec^. The molar ratios between SEPSECS, *E. coli* tRNA^Sec^, and other Sec synthetic enzymes within this synthetic system are unknown. Overexpressed archaeal PSTK would also compete for the *E. coli* tRNA^Sec^ substrate and could constrain how SEPSECS can interact with it, altering the aggregation phenomenon. Furthermore, the qualitative and indirect nature of the assay may obscure deficiencies in active SEPSECS•btRNA^Sec^. Similarly, murine SEPSECS with a truncated C-terminal tail also exhibited catalytic activity on par with the WT enzyme *in vitro* (32), but only in the presence of ∼2-fold molar excess of Sep-tRNA^Sec^ (32,55). Consequently, studies assessing whether other mammalian SEPSECS exhibit a similar dose-dependent aggregation behavior as the human enzyme are warranted. A comprehensive understanding of the mammalian C-terminus in Sec synthesis will require examining the function of α16 in a mammalian system, especially given that the sequence is unique to mammals. Finally, tRNA^Sec^ is scarce in human cells and SEPSECS is abundant over tRNA^Sec^ at a molar ratio of 13:1 (33), a level at which the aggregation behavior of the truncated enzyme presumably would be prominent. Broader studies on the origin and function of α16 may also provide greater insight regarding why autoantibodies in AIH patients target this region of SEPSECS and why that may be detrimental.

Given that the C-terminal tail is a more recent evolutionary innovation and is universally absent in archaeal SepSecS (30), it is unsurprising that the mammalian enzymes retain WT-like activity in its absence since the catalytic mechanism appears strongly conserved. MMP SepSecS displays a divergent substrate binding mechanism compared to the human enzyme as it lacks tRNA-binding groups and is unable to bind tRNA^Sec^ devoid of the phosphoseryl group. A detailed phylogenetic analysis highlighted considerable divergence between archaea and eukaryotes. The alpha helices critical for tRNA binding in the human enzyme are not faithfully conserved in MMP SepSecS. Moreover, the putative tRNA binding pocket in archaeal SepSecS is more acidic and lacks critical Arg and Lys residues for binding compared to mammalian orthologs. These results also help explain previous findings that showed that human SEPSECS maintained WT-like activity with mutations in α1, even when they significantly diminished binding affinity (34). If the system first evolved in the absence of tRNA recognition by α1, then it is unsurprising that mutations in α1 that diminish the binding affinity had a minimal effect on catalysis. Moreover, catalytic residues exhibited significantly higher conservation scores and sequence identity compared to residues involved in tRNA binding, suggesting the tRNA binding interface evolved more recently. Notably, MMP SepSecS did not appear as efficient as the human enzyme, suggesting the evolution in tRNA binding may have contributed to greater catalytic efficiency in the human enzyme. Overall, these findings show that human SEPSECS has evolved a more positively charged solvent-exposed surface that is conducive to tRNA recognition and provides electrostatic interactions to favor complex formation.

Yet questions concerning the mechanism of tRNA binding by archaeal SepSecS, the archaeal SepSecS•tRNA^Sec^ complex architecture, and its complex stoichiometry remain unanswered. Structural and binding studies of archaeal SepSecS in the presence of aminoacylated tRNA^Sec^ would help address these questions. Directly calculating the *k*_cat_ and *K*_M_ could explain how and/or if the evolutionary changes in the tRNA-binding interface affect enzyme kinetics, but the *in vitro* catalytic assay is currently unavailable.

Previous research has extensively characterized the nature of tRNA^Sec^ binding by human SEPSECS, revealing unique features crucial for substrate recognition, structural asymmetry, and organization of the active site (33,34,29). This study contextualizes that work within the broader framework of the evolutionary history of SepSecS, indicating that various elements of the human SEPSECS binding mechanism evolved over time to develop a more optimal tRNA binding interface. An intriguing explanation for these observations is the adaptation of a PLP Fold Type I-dependent enzyme over time to act on a tRNA substrate. SepSecS occupies separate phylogenetic branches than SelA and SepCysS (30), and thus has not evolved from another PLP Fold Type I-dependent enzyme acting on a tRNA-based substrate. Our data suggest that the evolution of the tRNA binding interface might have provided an advantage in generating a more efficient enzyme for Sec synthesis. Such changes in substrate binding may have then necessitated the emergence of a C-terminal helix to stabilize the SEPSECS•tRNA^Sec^ complex and perhaps provide a means for regulating the terminal step of Sec synthesis. Broader studies of SEPSECS from non-mammalian vertebrates may provide insight into what the evolutionary pressure was for the development of an α16 and why some branches of the vertebrate lineages lack an α16.

Ultimately, our study underscores the need for further investigations across non-mammalian species to address fundamental questions regarding the evolution of SepSecS and Sec synthesis. Previous work has indicated that PSTK co-evolved with SepSecS (56). How such coordinated changes between SepSecS and other enzymes in selenoprotein synthesis occurred could clarify how Sec metabolism and utilization adapted throughout evolution, particularly during the archaea-eukarya split, during which SepSecS underwent substantial modifications. Sec is often pivotal for redox enzymes (57,58) and for mitigating of oxidative inactivation (59,60). Given the profound transformations in cellular metabolism that accompanied the archaea–eukarya divide (61), grasping how Sec synthesis and usage altered during these broader metabolic changes could answer long-standing questions about the evolutionary advantages of Sec expansion into the genetic code and its incorporation into proteins (59,60,62).

## Supplementary Material

gkae875_Supplemental_Files

## Data Availability

The data underlying this article are available within the article and in its associated online supplementary material.

## Notes

Present address: Jennifer Castillo Suchkou, Rush Medical College, Rush University Medical Center, Chicago, IL 60612, USA.

Present address: Rachel L. French, Agilent Technologies Inc., Santa Clara, CA, USA.

Present address: Malgorzata Dobosz-Bartoszek, Cytiva, Marlborough, MA, USA.

Present address: Kaitlyn A. Kiernan, Department of Molecular Biosciences, University of Texas at Austin, Austin, TX 78712, USA.

Present address: Miljan Simonović, National Institutes of Health, National Institute of General Medical Sciences, Bethesda, MD 20892, USA.
