# Supplementary material for: Human selenocysteine synthase, SEPSECS, has evolved to optimize binding of a tRNA-based substrate
Source: Nucleic Acids Res. 2024 Oct 10;52(21):13368–85. doi: 10.1093/nar/gkae875 (PMC11602143; doi:10.1093/nar/gkae875)
Supplement: gkae875_Supplemental_Files [file gkae875_supplemental_files.zip › NAR_SOM.pdf]

Supporting Online Materials

Human selenocysteine synthase, SEPSECS, has evolved to optimize binding of a tRNA-based substrate

Anupama K. Puppala[1*], Dylan Sosa[2], Jennifer Castillo Suchkou[1$], Rachel L. French[1†], Malgorzata Dobosz-Bartoszek[1], Kaitlyn A. Kiernan[1§], Miljan Simonović[1&*]

[1] Department of Biochemistry and Molecular Genetics, University of Illinois at Chicago, Chicago, Illinois 60607, USA
[2] Department of Ecology & Evolution, University of Chicago, Chicago, IL 60637, USA

* Corresponding authors:

Miljan Simonović, PhD

National Institutes of Health

National Institute of General Medical Sciences

Bethesda, MD 20892

USA

Email: miljan.simonovic@nih.gov

Anupama Puppala, PhD

Department of Biochemistry and Molecular Genetics

University of Illinois at Chicago

900 S. Ashland Ave., 1354 MBRB

Chicago, IL 60607

U.S.A.

Email: puppala2@uic.edu

[$] Present address: Rush Medical College, Rush University Medical Center, Chicago, IL 60612, USA
[†] Present Address: Agilent Technologies Inc., Santa Clara, CA, USA
[#] Present Address: Cytiva, Marlborough, MA, USA
[§] Present Address: Department of Molecular Biosciences, University of Texas at Austin, Austin, Texas, 78712
[&] Present address: National Institutes of Health, National Institute of General Medical Sciences, Bethesda, MD 20892, USA

SUPPLEMENTAL TABLES AND FIGURES

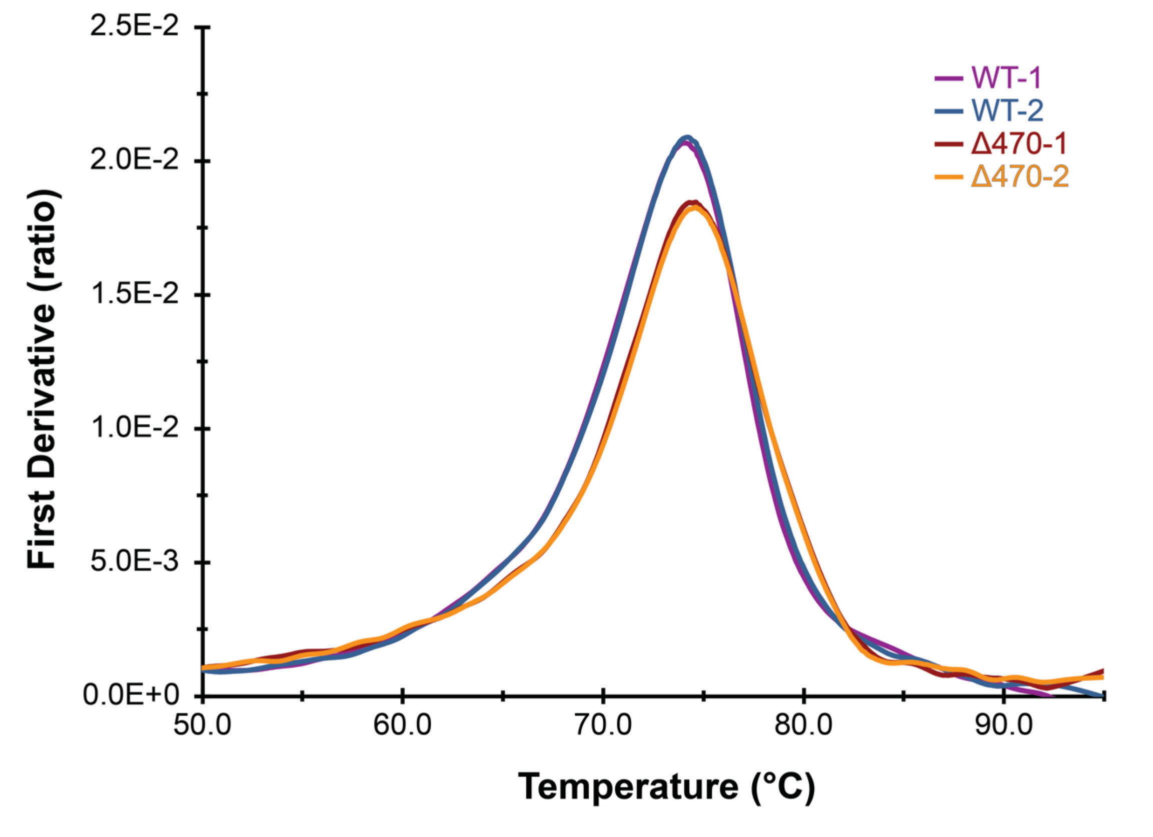

Figure S1. WT and Δ470 SEPSECS possess similar unfolding trajectories. A plot of the first derivative of the intrinsic fluorescence ratio (350 nm/330 nm) versus temperature illustrates that the WT (blue/purple traces) and Δ470 (orange/red traces) enzymes have similar unfolding profiles.

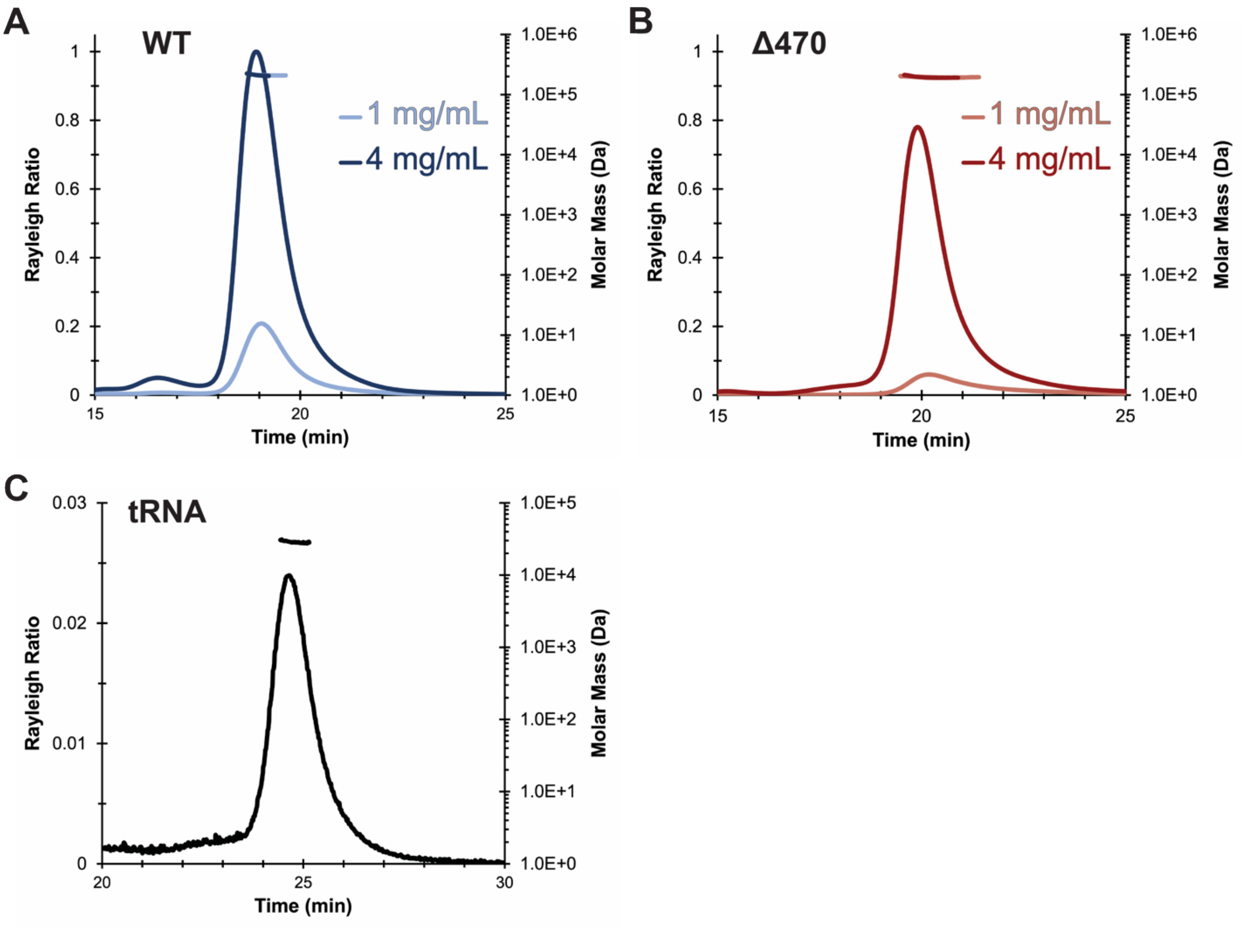

Figure S2. WT and Δ470 SEPSECS both form a stable, tetrameric species. Multi-angle light scattering (MALS) traces of **(A)** WT SEPSECS (blue traces) and **(B)** Δ470 SEPSECS (red traces) revealed a single, monodisperse species with a horizontal molar mass distribution. Molecular weight determination using these traces indicated a tetrameric species for both WT and Δ470 SEPSECS. The molecular weight determinations and polydispersity did not change with an increase in the concentration for either species. **(C)** Light scattering trace of unacylated human $tRNA^{Sec}$ (black trace) shows $tRNA^{Sec}$ adopts a single monomeric species.

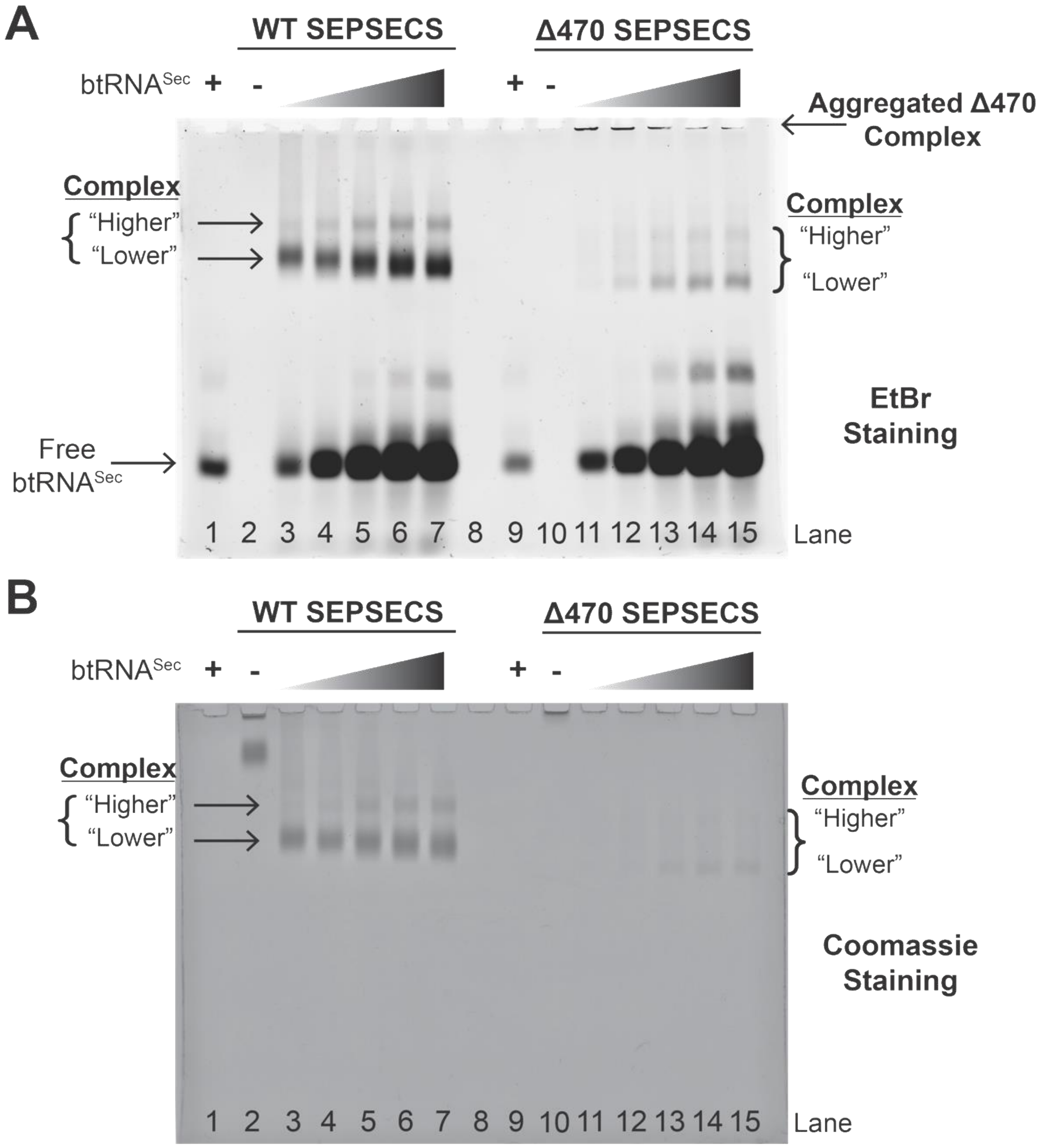

Figure S3. Δ470 SEPSECS exhibits an increased tendency to form higher-order species with bacterial tRNA^{Sec}. (A) EtBr staining shows the change in migration of bacterial tRNA^{Sec} (btRNA^{Sec}) in a titration series upon binding either human WT or Δ470 SEPSECS. Binding of btRNA^{Sec} to WT or Δ470 SEPSECS formed some soluble complex, as indicated by the "higher" and "lower" EtBr-staining bands with reduced electrophoretic mobility. However, binding to the Δ470 SEPSECS also generates a higher-order species at all tested molar ratios, as indicated by staining within the well. **(B)** Coomassie staining of the same gel confirms the presence of protein in the complex. Lane 1 - free btRNA^{Sec}, Lane 2 - WT, Lane 3 - 4:1 WT: btRNA^{Sec}, Lane 4 - 4:2 WT: btRNA^{Sec}, Lane 5 - 4:4 WT: btRNA^{Sec}, Lane 6 - 4:6 WT: btRNA^{Sec}, Lane7 - 4:8 WT: btRNA^{Sec}, Lane 8 – empty, Lane 9 - free btRNA^{Sec}, Lane 10 - Δ470, Lane 11 - 4:1 Δ470: btRNA^{Sec}, Lane 12 - 4:2 Δ470: btRNA^{Sec}, Lane 13 - 4:4 Δ470: btRNA^{Sec}, Lane 14 - 4:6 Δ470: btRNA^{Sec}, Lane 15 - 4:8 Δ470: btRNA^{Sec}.

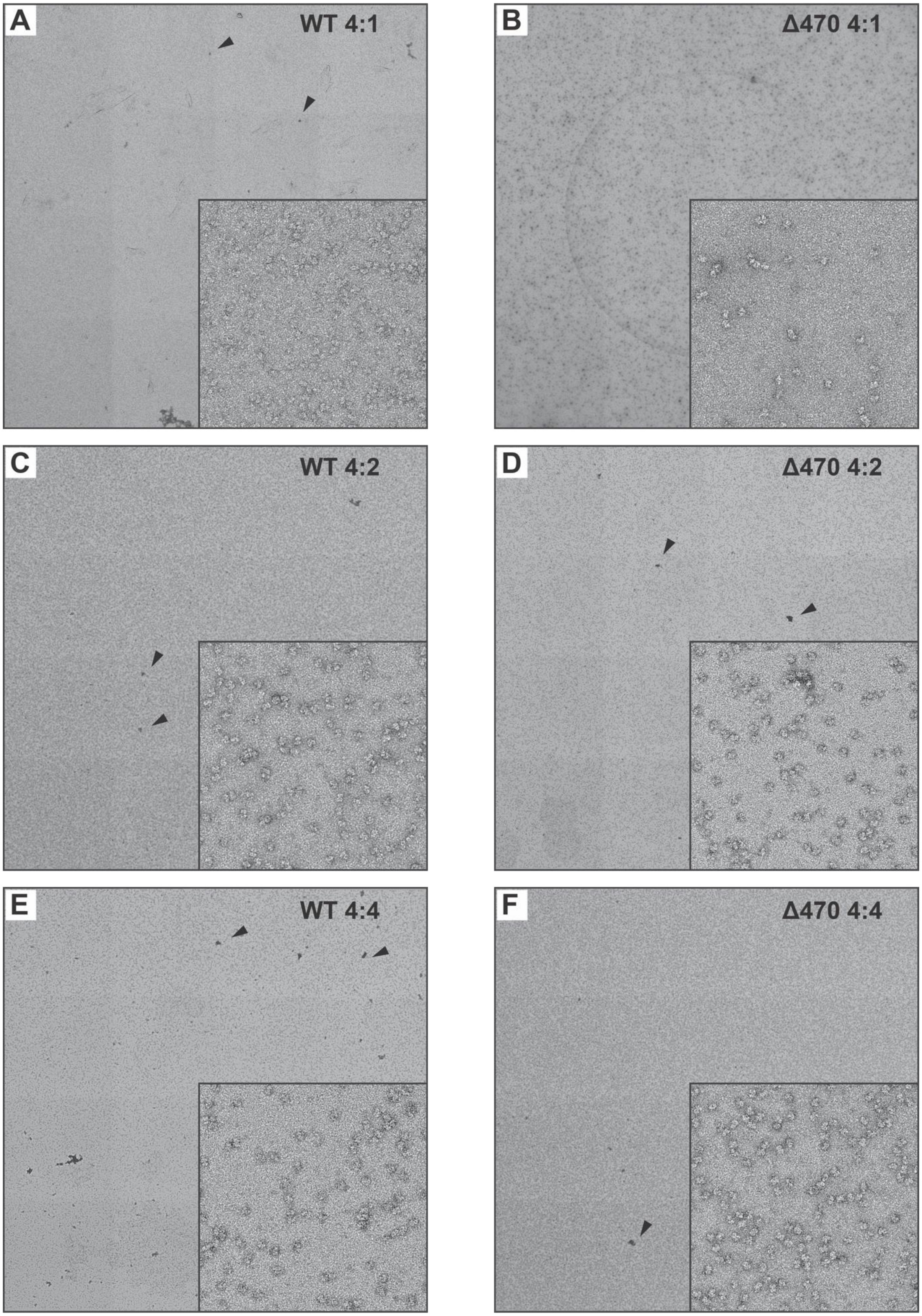
A
WT 4:1
B
Δ470 4:1
C
WT 4:2
D
Δ470 4:2
E
WT 4:4
F
Δ470 4:4

Figure S4. Negative staining shows aggregation of the Δ470 SEPSECS•tRNA^{Sec} complex at low tRNA^{Sec} concentrations. For each sample, the large box is a montage of 4 x 4 electron micrograph grids taken at 25,000x, while the insets are higher magnification images taken at 49,000x from within the larger panel. **(A, C, E)** WT SEPSECS particles look similar across the range of tRNA^{Sec} concentrations (from 1 to 4 tRNAs per tetramer) with little apparent aggregated stain (arrowheads). **(B)** Δ470 SEPSECS exhibits ubiquitous aggregation in the low magnification image as indicated by the widespread black particles due to aggregated stain. The inset shows far fewer tetrameric particles. **(D & F)** Δ470 SEPSECS particles resembles the WT enzyme at higher molar ratios of tRNA^{Sec} (from 2 to 4 tRNAs per tetramer), with little apparent aggregated stain (arrowheads).

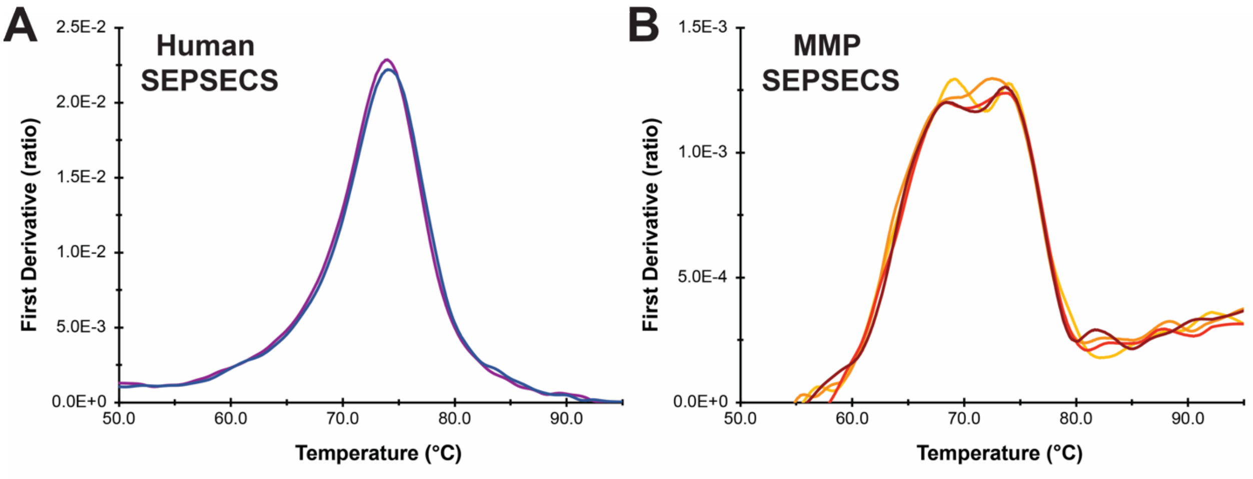

Figure S5. ***M. maripaludis*** **SepSecS is a folded enzyme.** A plot of the first derivative of the fluorescence ratio (350 nm/330 nm) versus temperature for human SEPSECS and MMP SepSecS shows that they have different unfolding profiles yet possess inflection temperatures in a similar range.

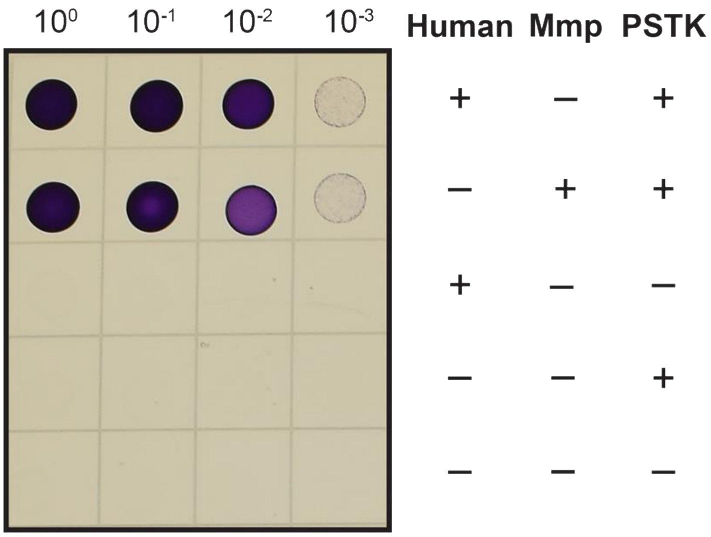

Figure S6. The *E. coli*-based functional assay suggests archaeal SepSecS is less efficient than human SEPSECS. Co-expression of human SEPSECS and *M. jannaschii* PSTK allows Δ*selA* JS2(DE3) cells to synthesize formate dehydrogenase and reduce benzyl viologen (BV) to its purple form. MMP SepSecS exhibits similar levels of BV reduction as the human enzyme at the 10^{0} and 10^{-1} dilutions, though it appears less effective at BV reduction at the 10^{-2} dilution. Experiment was performed in duplicate with one representative result shown.

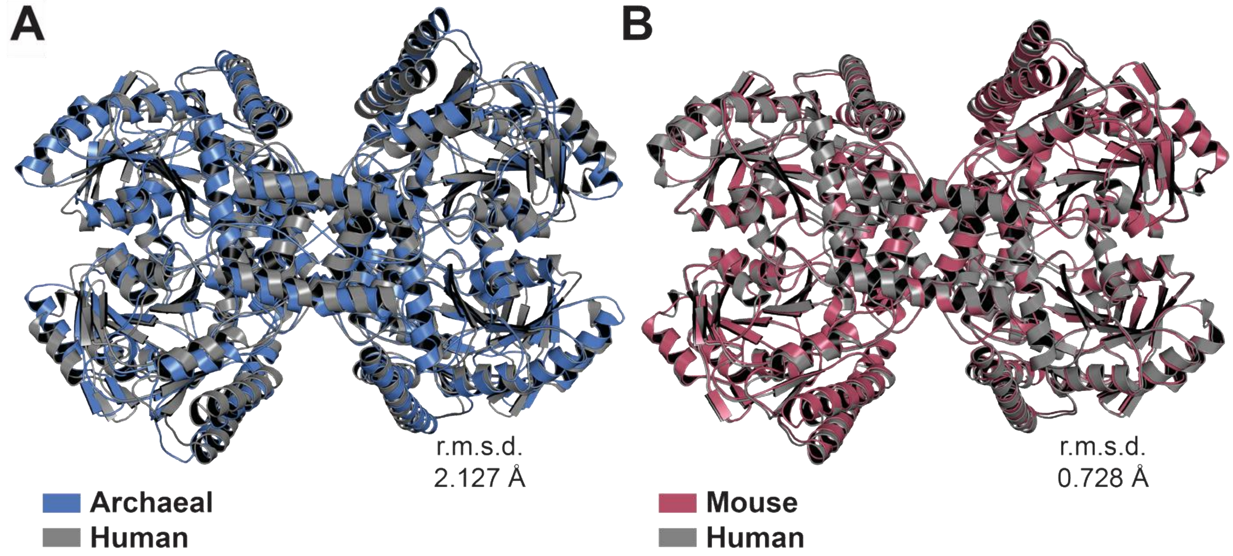

Figure S7. Human and murine SEPSECS and archaeal SepSecS all adopt a similar tetrameric architecture. (A) Superimposition of the holo structures of human SEPSECS (PDBID 7L1T; gray cartoon) and archaeal SepSecS (PDBID 2Z67; blue cartoon); RMSD of 2.127 Å over 8,789/10,335 aligned atoms. **(B)** Superimposition of the holo structures of human SEPSECS and murine SEPSECS (PDBID 3BC8; red cartoon); RMSD of 0.728 Å over 12,217/13,219 aligned atoms.

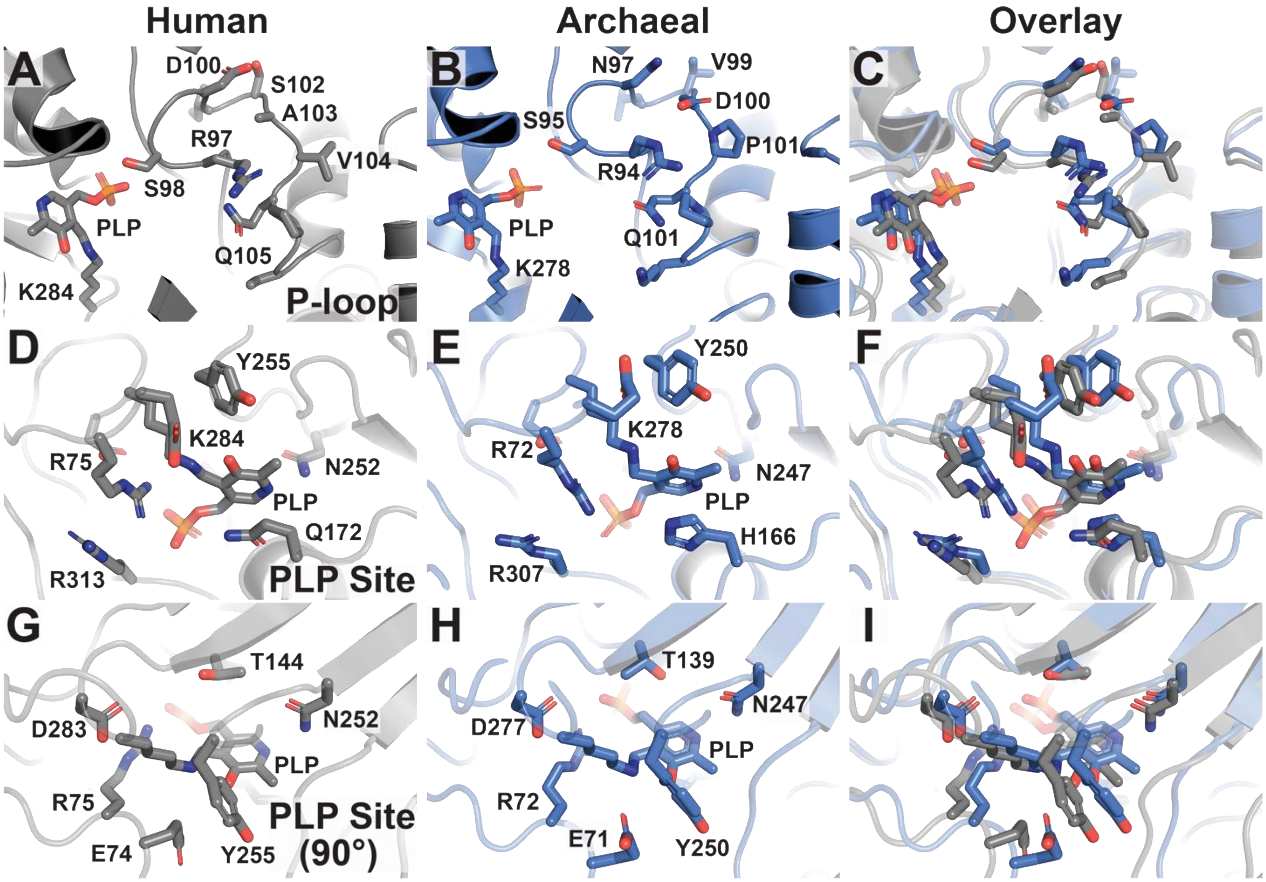

Figure S8. Human SEPSECS and MMP SepSecS have similarly organized catalytic sites. (A-C) The phosphate-binding loop for binding phosphorylated reaction substrates is similarly organized in both human SEPSECS (PDBID 7L1T; gray cartoon) and archaeal SepSecS (PDBID 2Z67; blue cartoon). (**D-F)** Amino acids that anchor the PLP co-factor to support electrophilic catalysis are strongly conserved between the archaeal and human enzymes. **(G-I)** Same as **(D-F)** with the view rotated 90° around the axis perpendicular to the plane of view.

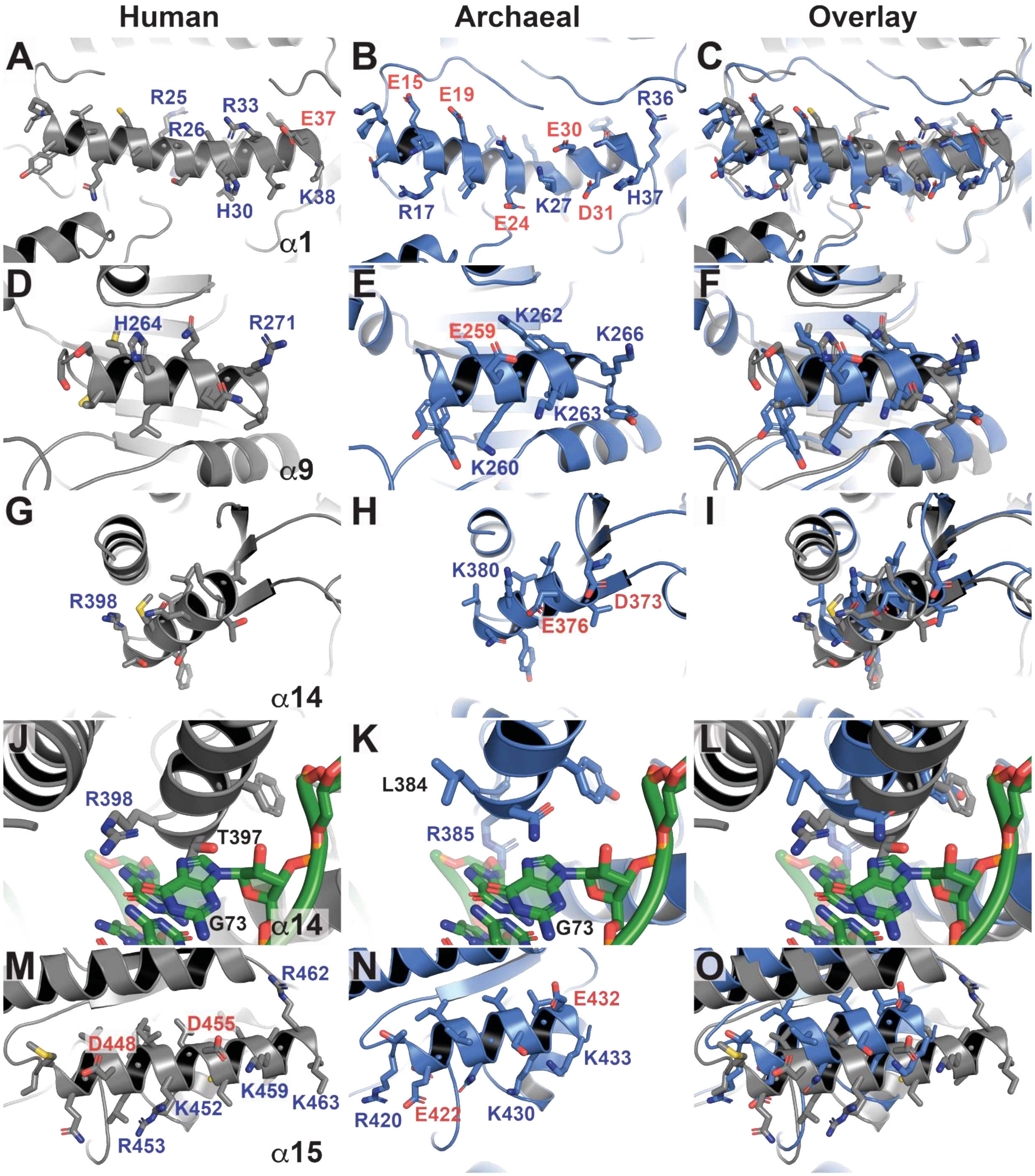

Figure S9. Alpha helices in human SEPSECS that mediate tRNA binding are poorly conserved in MMP SepSecS. Comparison of the putative binding helices and their charged residues from human SEPSECS (PDBID 7L1T; gray cartoon) and MMP SepSecS (PDBID 2Z67; blue cartoon). **(A-C)** α1 in MMP SepSecS features more acidic (red) residues than the human enzyme. **(D-F)** α9 exhibits the most structural similarity between the two enzymes. **(G-I)** α14 is oriented differently and features more acidic residues than MMP SepSecS. **(J-L)** Modeling human $tRNA^{Sec}$ (PDBID 7MDL; green cartoon) into the putative binding site reveals that MMP SepSecS lacks an equivalent residue to Arg398 to engage G73. **(M-O)** MMP SepSecS has a shorter α15 lacking several basic (blue) residues found in human SEPSECS.

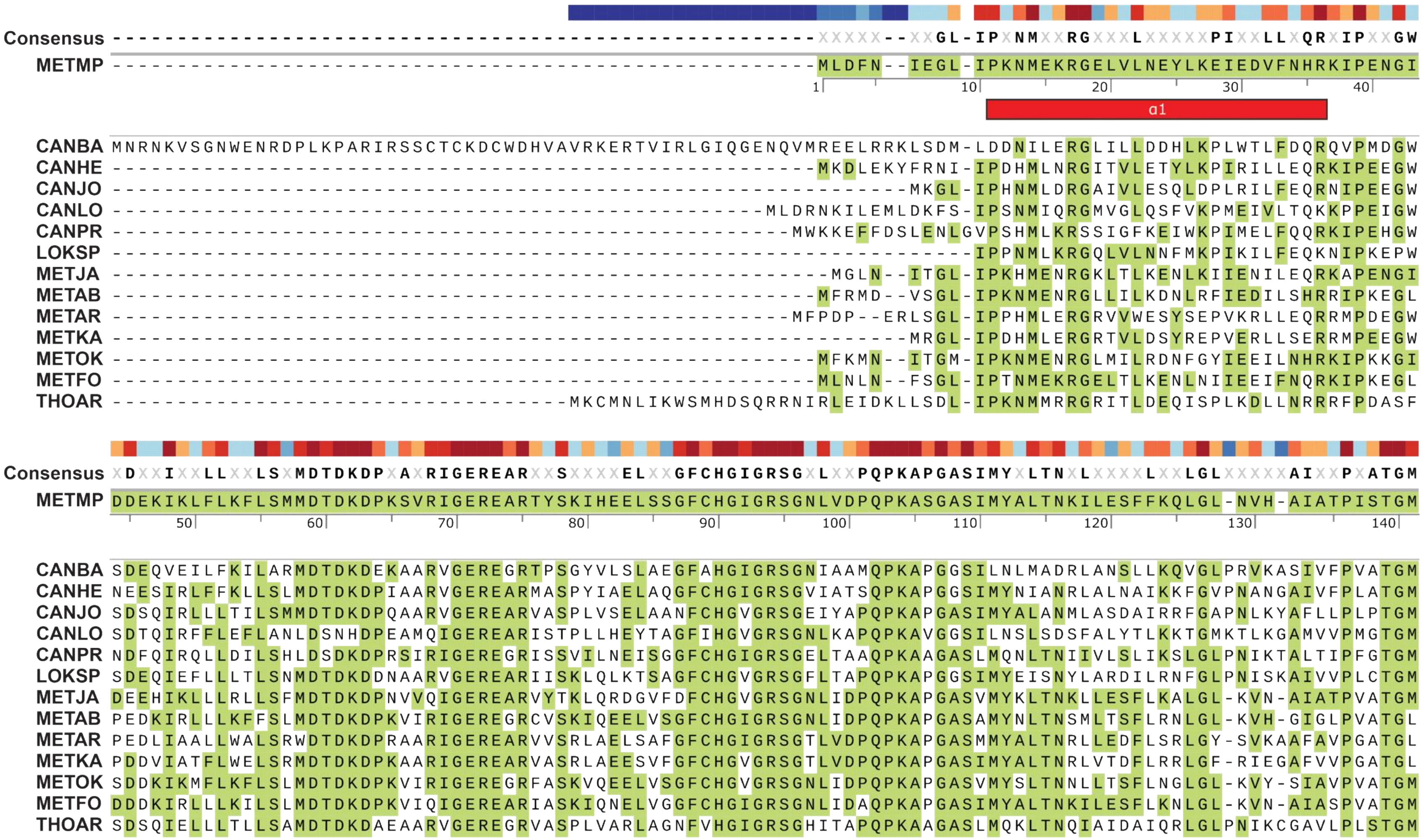
Consensus ---XXXXX--XXGL-IPXNMXXRGXXXLXXXXXPIXXLLXQRXIPXXGW
METMP ---MLDFN--IEGL-IPKNMEKRGELVLNEYLKEIEDVFNHRKIPENGI
1 10 20 30 40
α1
CANBA MNRNKVSGNWENRDPLKPARIRSSCTCKDCWDHVAVRKERTVIRLGIQGENQVMREELRRKLSDM-LDDNILERGLILLDDHLKPLWTLFDQRQVPMDGW
CANHE ---MKDLEKYFRNI-IPDHMLNRGITVLETYLKPIRILLEQRKIPEEGW
CANJO ---MKGL-IPHNMLDRGAIVLESQLDPLRILFEQRNIPEEGW
CANLO ---MLDRNKILEMLDKFS-IPSNMIQRGMVGLQSFVKPMEIVLTQKKPPEIGW
CANPR ---MWKKEFFDSLENLGVPSHMLKRSSIGFKEIWKPIMELFQQRKIPEHGW
LOKSP ---IPPNMLKRGQLVLNNFMKPIKILFEQKNIPKEPW
METJA ---MGLN--ITGL-IPKHMENRGKLTLKENLKIIENILEQRKAPENGI
METAB ---MFRMD--VSGL-IPKNMENRGLLILKDNLRFIEDILSHRRIPKEGL
METAR ---MFPDP--ERLSGL-IPPHMLERGRVVWESYSEPVKRLLEQRRMPDEGW
METKA ---MRGL-IPDHMLERGRTVLDSYREPVERLLSERRMPEEGW
METOK ---MFKMN--ITGM-IPKNMENRGLMILRDNFGYIEEILNHRKIPKKGI
METFO ---MLNLN--FSGL-IPTNMEKRGELTLKENLNIIEEIFNQRKIPKEGL
THOAR ---MKCMNLIKWSMHDSQRRNIRLEIDKLLSDL-IPKNMMRRGRITLDEQISPLKDLLNRRRFPDASF
Consensus XDXXIXXLLXXLSXMDTDKDPXAXRIGEREARXXSXXXXELXXGFCHGIGRSGXLXXPQPKAPGASIMYXLTNXLXXXXLXXLGLXXXXXAIXXPXATGM
METMP DDEKIKLFLKFLSMMDTDKDPKSVRIGEREARTYSKIHEELSSGFCHGIGRSGNLVDPQPKASGASIMYALTNKILESFFKQLGL-NVH-AIATPISTGM
50 60 70 80 90 100 110 120 130 140
CANBA SDEQVEILFKILARMDTDKDEKAARVGEREGRTPSGYVLSLAEGFAHGIGRSGNIAAMQPKAPGGSILNLMADRLANSLLKQVGLPRVKASIVFPVATGM
CANHE NEESIRLFFKLLSLMDTDKDPIAARVGEREARMASPYIAELAQGFCHGIGRSGVIATSQPKAPGGSIMYNIANRLALNAIKKFGVPNANGAIVFPLATGM
CANJO SDSQIRLLLTILSMMDTDKDPQAARVGEREARVASPLVSELAANFCHGVGRSGEIYAPQPKAPGASIMYALANMLASDAIRRFGAPNLKYAFLLPLPTGM
CANLO SDTQIRFFLEFLANLDSNHDPEAMQIGEREARISTPLLHEYTAGFIHGVGRSGNLKAPQPKAVGGSILNSLSDSFALYTLKKTGMKTLKGAMVVPMGTGM
CANPR NDFQIRQLLDILSHLDSDKDPRSIRIGEREGRISSVILNEISGGFCHGIGRSGELTAAQPKAAGASLMQNLTNIIVLSLIKSLGLPNIKTALTIPFGTGM
LOKSP SDEQIEFLLLTLSNMDTDKDDNAARVGEREARIISKLQLKTSAGFCHGVGRSGFLTAPQPKAPGGSIMYEISNYLARDILRNFGLPNISKAIVVPLCTGM
METJA DEEHIKLLLRLLSFMDTDKDPNVVQIGEREARVYTKLQRDGVFDFCHGVGRSGNLIDPQPKAPGASVMYKLTNKLLESFLKALGL-KVN-AIATPVATGM
METAB PEDKIRLLLKFFSLMDTDKDPKVIRIGEREGRCVSKIQEELVSGFCHGIGRSGNLIDPQPKAPGASAMYNLTNSMLTSFLRNLGL-KVH-GIGLPVATGL
METAR PEDLIAALLWALSRWDTDKDPRAARIGEREARVVSRLAELSAFGFCHGIGRSGTLVDPQPKAPGASMMYALTNRLLEDFLSRLGY-SVKAAFAVPGATGL
METKA PDDVIATFLWELSRMDTDKDPKAARIGEREARVASRLAEESVFGFCHGVGRSGTLVDPQPKAPGASIMYALTNRLVTDFLRRLGF-RIEGAFVVPGATGL
METOK SDDKIKMFLKFLSLMDTDKDPKVIRIGEREGRFASKVQEELVSGFCHGVGRSGNLIDPQPKAPGASVMYSLTNNLLTSFLNGLGL-KVY-SIAVPVATGM
METFO DDDKIRLLLKILSLMDTDKDPKVIQIGEREARIASKIQNELVGGFCHGIGRSGNLIDAQPKAPGASIMYALTNKILESFLKNLGL-KVN-AIASPVATGM
THOAR SDSQIELLLTLLSAMDTDKDAEAARVGEREGRVASPLVARLAGNFVHGIGRSGHITAPQPKAAGASLMQKLTNQIAIDAIQRLGLPNIKCGAVLPLSTGM

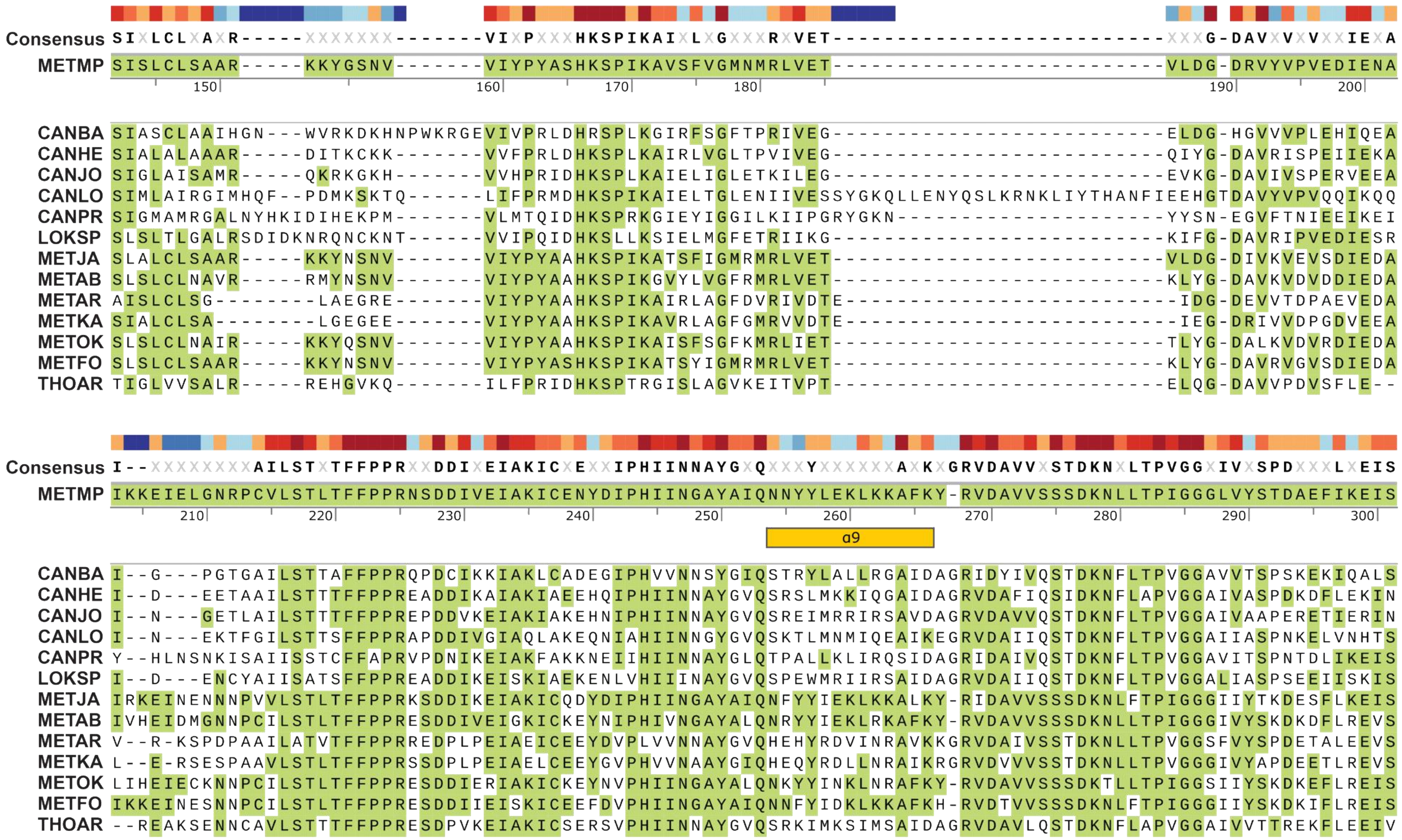

Consensus
METMP
150
160
170
180
190
200
CANBA
CANHE
CANJO
CANLO
CANPR
LOKSP
METJA
METAB
METAR
METKA
METOK
METFO
THOAR
Consensus
METMP
210
220
230
240
250
260
270
280
290
300
α9
CANBA
CANHE
CANJO
CANLO
CANPR
LOKSP
METJA
METAB
METAR
METKA
METOK
METFO
THOAR

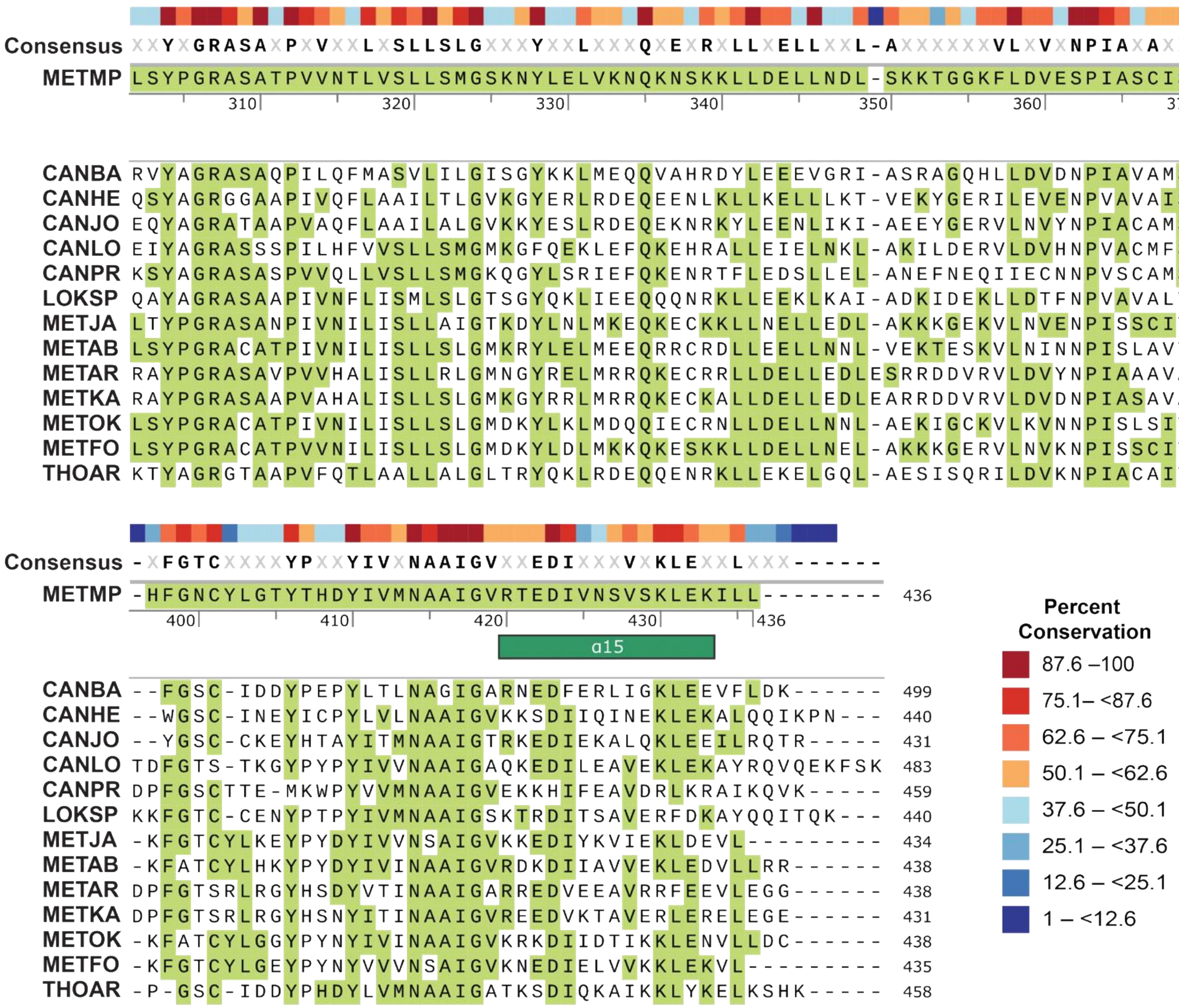

Figure S10. Conservation across archaeal SepSecS orthologs. Multiple sequence alignment of archaeal SepSecS. Colored blocks above the consensus sequence indicate the relative conservation of that position according to the method of Valder. The putative tRNA binding helices are annotated below the METMP sequence (MMP SepSecS) according to PDBID 2Z67. Positions that match the METMP sequence are highlighted in green. *Candidatus Bathyarchaeota* (CANBA), *Candidatus Helarchaeota* (CANHE), *Candidatus Jordarchaeia* (CANJO), *Candidatus Lokiarchaeota* (CANLO), *Candidatus Prometheoarchaeum* (CANPR), *Lokiarchaeum sp.* (LOKSP), *Methanocaldococcus jannaschii* (METJA), *Methanofervidicoccus abyssi* (METAB), *Methanopyri archaeon* (METAR), *Methanopyrus kandleri* (METKA), *Methanothermococcus okinawensis* (METOK), *Methanotorris formicicus* (METFO), *Thorarchaeota archaeon* (THOAR).

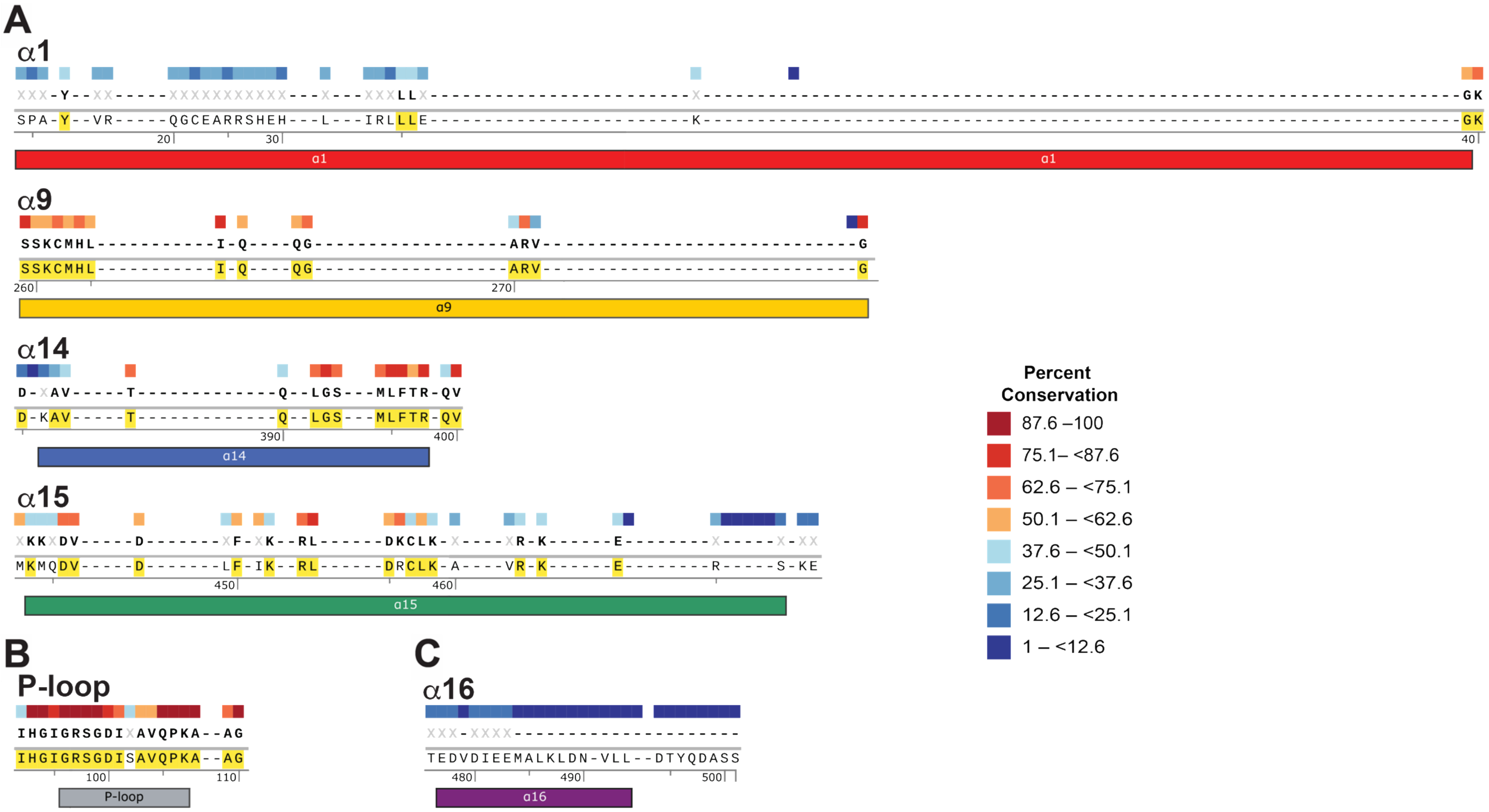

Figure S11. Conservation of human SEPSECS elements. The human SEPSECS amino acid sequence (bottom sequence) relative to the consensus sequence (top sequence) derived from an alignment of 874 sequences across archaea and eukarya. Colored blocks above the consensus sequence indicate the relative conservation at each position, based on the Valder method. Positions where the human and consensus sequences match are highlighted in yellow **(A)** Among the tRNA binding helices, α9 and α14 have the strongest conservation, α15 has some conserved residues, while α1 is highly variable. **(B)** Conversely, catalytic regions, such as the P-loop, exhibit strong conservation. **(C)** α16 residues show minimal conservation. Helices α1, α9, α14, and α15 are annotated according to PDBID 7L1T and α16 according to PDBID 7MDL.

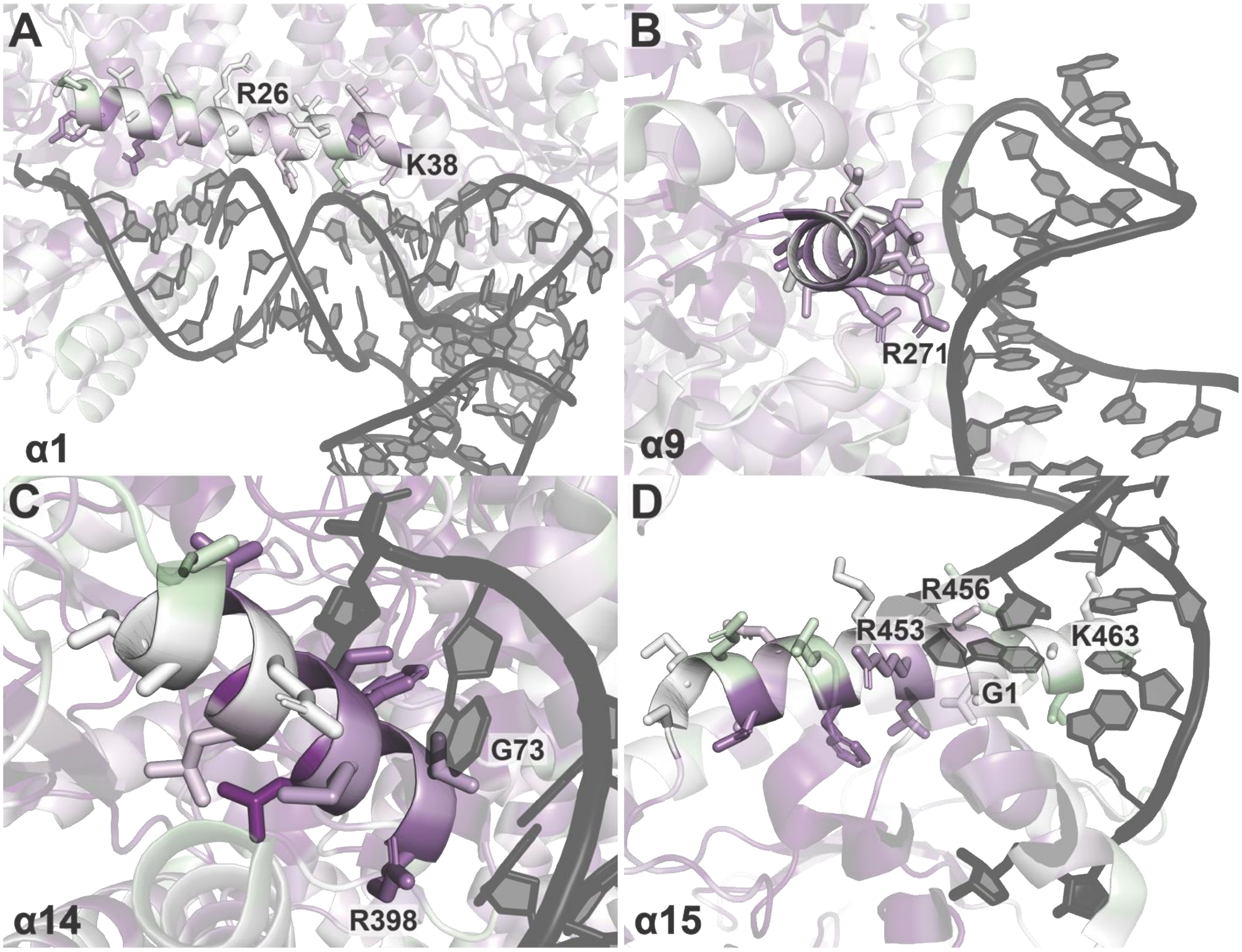

Figure S12. Consurf-generated conservation scores for the human SEPSECS alpha helices that comprise the tRNA-binding pocket. Helices α1 **(A)**, α9 **(B)**, α14 **(C)**, and α15 **(D)**.

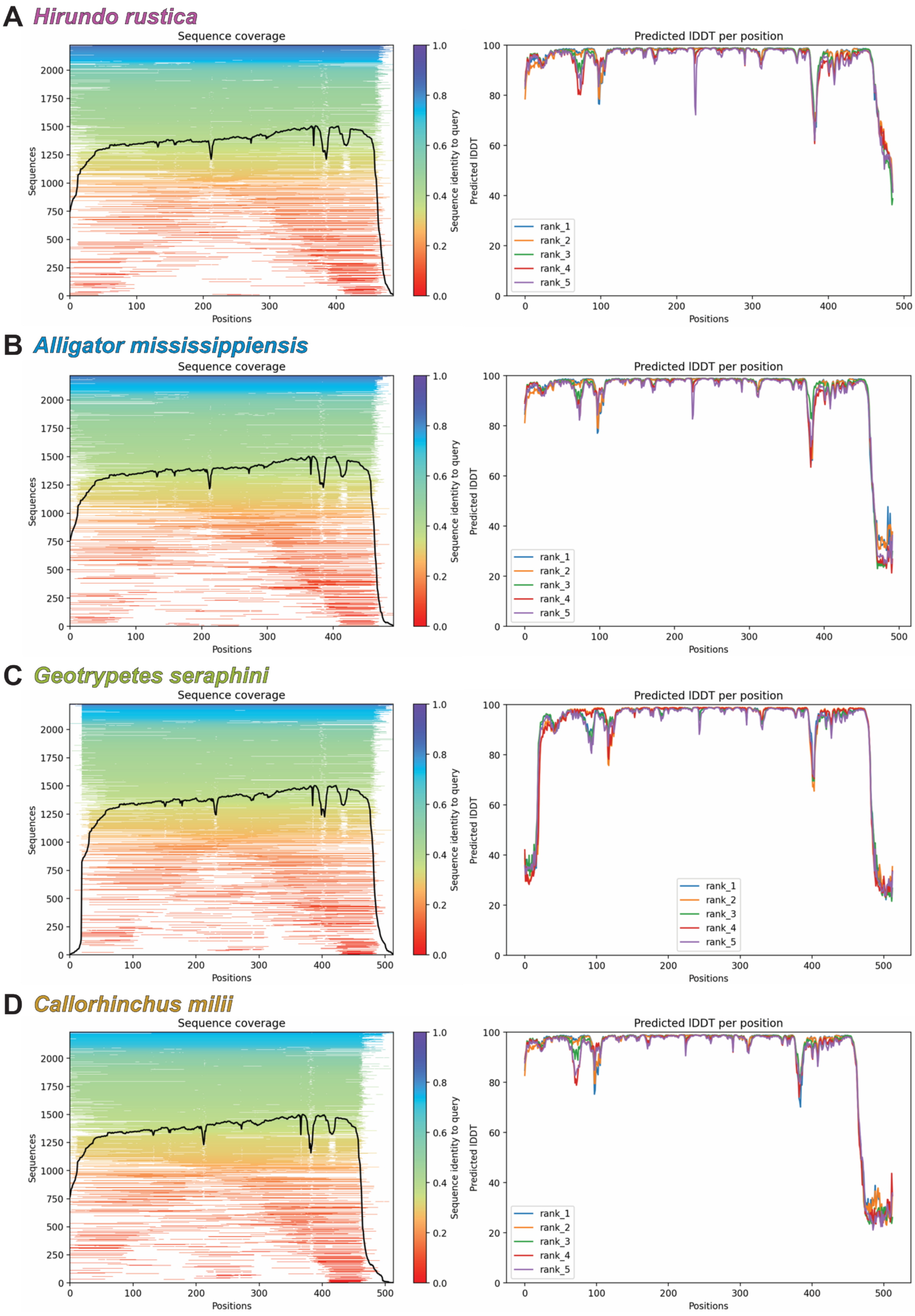
A Hirundo rustica
B Alligator mississippiensis
C Geotrypetes seraphini
D Callorhinchus milii
Sequence coverage
Sequences
Positions
Sequence identity to query
Predicted lDDT per position
Predicted lDDT
rank_1
rank_2
rank_3
rank_4
rank_5

Figure S13. Low sequence coverage for the extended C-terminus of SEPSECS orthologs contributes to low-confidence predictions. The sequence coverage (left) drops dramatically for the C-terminal extension in all non-mammalian, vertebrate orthologs of SEPSECS: **(A)** *Hirundo rustica* (avian), **(B)** *Alligator mississippiensis* (reptilian), **(C)** *Geotrypetes seraphini* (amphibian), **(D)** *Callorhinchus milii* (fish). The predicted local distance difference test (pLDDT; right), indicating the confidence in the local structure prediction, is poor (<50%) for the C-terminus. .

Table S1. MALS-derived molecular weights of human SEPSECS species.

WT SEPSECS•tRNA^{Sec}					
**(SEPSECS : tRNA^{Sec}) ratio**	**4:1**	**4:2**	**4:4**	**4:6**	**4:8**
**MW (kDa ± %uncertainty)**	238 ± 3.7%	242 ± 2.9%	243 ± 2.9%	249 ± 3.1%	248 ± 2.8%
**Polydispersity**	1.00 ± 5.2%	1.00 ± 4.1%	1.00 ± 4.2%	1.00 ± 4.4%	1.00 ± 4.0%
Δ470 SEPSECS•tRNA^{Sec}					
**(SEPSECS : tRNA^{Sec}) ratio**	**4:1**[a]	**4:2**	**4:4**	**4:6**	**4:8**
**MW (kDa ± %uncertainty)**	**ND**	**N/A**	**N/A**	**N/A**	**N/A**
**Polydispersity**	**ND**	1.00 ± 4.0%	1.01 ± 18%	1.02 ± 18%	1.01 ± 19%

[a] Not determined since aggregation of the sample resulted in no discernible signal.

Table S2. Thermal unfolding parameters for MMP SepSecS.

Sample	T_i1[a]	T_i2[a]	Initial Ratio[b]	Δ Ratio[c]
Human SEPSECS	74.0 ± 0.1	N/A	0.594 ± 0.001	0.262 ± 0.002
MMP SepSecS	69.1	73.4 ± 0.6	0.259 ± 0.000	0.021 ± 0.001

[a] T_i is the inflection temperature of the unfolding transition in the signal of the 350 nm/330 nm ratio.
[b] Initial Ratio is the value of the ratio of 350 nm/330 nm at the beginning of the measurement.
[c] Δ Ratio is the difference between the ratio at the beginning and at the end of the thermal profile.

Table S3. Conservation of notable amino acid residues in human SEPSECS

Amino Acid	Role	Conservation Score[a]	Identity[b]	Amino Acid	Role	Conservation Score[a]	Identity[b]
Arg26	Binding	0.28	0.33	Glu477	α16	0.15	0.21
Lys38	Binding	0.40	0.30	Asp478	α16	0.16	0.22
Lys40	Binding	0.67	0.66	Val479	α16	0.06	0.07
Glu74	Catalysis	0.92	0.98	Asp480	α16	0.14	0.24
Arg75	Catalysis	0.93	0.98	Ile481	α16	0.13	0.22
Gly96	P-loop	0.94	0.99	Glu482	α16	0.16	0.38
Arg97	P-loop	0.94	0.98	Glu483	α16	0.13	0.23
Ser98	P-loop	0.93	0.98	Met484	α16	0.04	0.12
Gly99	P-loop	0.92	0.95	Ala485	α16	0.02	0.12
Asp100	P-loop	0.81	0.82	Leu486	α16	0.02	0.14
Ile101	P-loop	0.73	0.65	Lys487	α16	0.02	0.13
Ser102	P-loop	0.47	0.30	Leu488	α16	0.02	0.15
Ala103	P-loop	0.62	0.62	Asp489	α16	0.02	0.15
Val104	P-loop	0.56	0.49	Asn490	α16	0.02	0.14
Gln105	P-loop	0.94	0.99	Val491	α16	0.01	0.14
Pro106	P-loop	0.95	0.99	Leu492	α16	0.01	0.16
Lys107	P-loop	0.93	0.98	Leu493	α16	0.01	0.13
Thr144	Catalysis	0.94	0.98				
Gly145	Catalysis	0.95	0.98				
Gln172	Catalysis	0.92	0.94				
Cys175	Catalysis	0.82	0.84				
Asn252	Catalysis	0.97	0.98				
Tyr255	Catalysis	0.97	0.97				
Arg271	Binding	0.66	0.64				
Asp283	Catalysis	0.98	0.98				
Lys284	Catalysis	0.98	0.98				
Arg313	Catalysis	0.94	0.89				
Ser393	Catalysis	0.73	0.76				
Phe396	Catalysis	0.81	0.83				
Thr397	Catalysis	0.54	0.53				
Arg398	Binding/Catalysis	0.79	0.84				
Gln399	Binding	0.43	0.40				
Arg453	Binding	0.69	0.72				
Arg456	Binding	0.63	0.67				
Lys463	Binding	0.45	0.51				

[a] *The identity is the pairwise identity over the alignment as calculated by Geneious Prime.*
[b] *The conservation score was calculated by SnapGene using the method of Valder.*
Residues with a role in catalysis are colored red and those involved in binding are colored blue.
