# Supplementary material for: Human selenocysteine synthase, SEPSECS, has evolved to optimize binding of a tRNA-based substrate
Source: Nucleic Acids Res. 2024 Oct 10;52(21):13368–85. doi: 10.1093/nar/gkae875 (PMC11602143; doi:10.1093/nar/gkae875)
Supplement: gkae875_Supplemental_Files [file FigS14_RectangularTree.pdf › FigS14_RectangularTree.pdf]

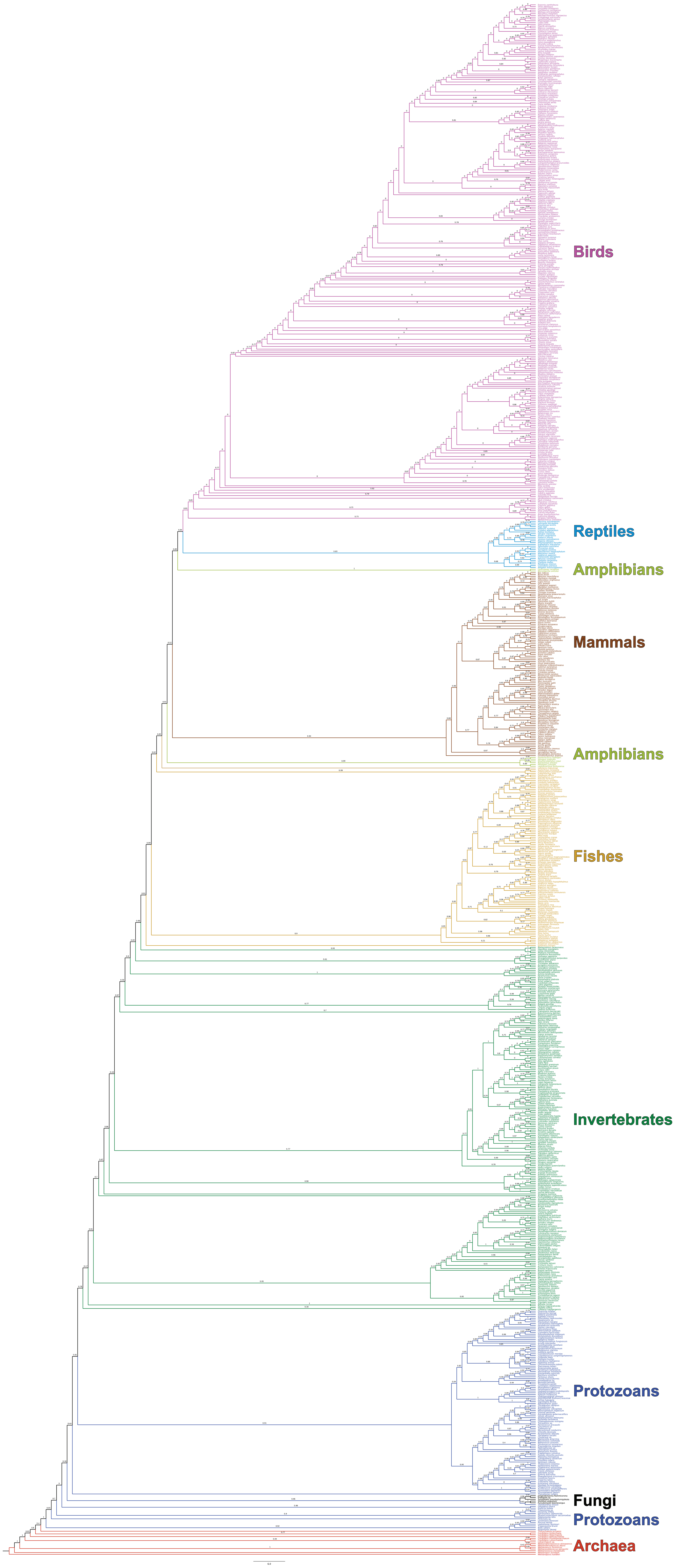
Birds
Reptiles
Amphibians
Mammals
Amphibians
Fishes
Invertebrates
Protozoans
Fungi
Protozoans
Archaea
